# Human cytomegalovirus infection impairs neural differentiation via repressing sterol regulatory element binding protein 2-mediated cholesterol biosynthesis

**DOI:** 10.1007/s00018-024-05278-0

**Published:** 2024-07-06

**Authors:** Jianming Li, Jingxuan Sun, Mingyi Xu, Lei Yang, Ning Yang, Jingui Deng, Yanping Ma, Ying Qi, Zhongyang Liu, Qiang Ruan, Yao Liu, Yujing Huang

**Affiliations:** 1 Virology Laboratory, Shengjing Hospital of China Medical University, Shenyang, Liaoning China; 2 Department of Pediatrics, Shengjing Hospital of China Medical University, Shenyang, Liaoning China; 3 Departments of Obstetrics and Gynecology, Shengjing Hospital of China Medical University, Shenyang, Liaoning China; 4 Department of Microorganism Laboratory, Shenyang Center for Disease Control and Prevention, Shenyang, Liaoning China; 5 Department of Pediatric Dentistry, School and Hospital of Stomatology, China Medical University, Shenyang, Liaoning China; 6 Liaoning Provincial Key Laboratory of Oral Diseases, Shenyang, Liaoning China

**Keywords:** Human cytomegalovirus, Neural differentiation, Cholesterol, SREBP2

## Abstract

Congenital human cytomegalovirus (HCMV) infection is a major cause of abnormalities and disorders in the central nervous system (CNS) and/or the peripheral nervous system (PNS). However, the complete pathogenesis of neural differentiation disorders caused by HCMV infection remains to be fully elucidated. Stem cells from human exfoliated deciduous teeth (SHEDs) are mesenchymal stem cells (MSCs) with a high proliferation and neurogenic differentiation capacity. Since SHEDs originate from the neural crest of the early embryonic ectoderm, SHEDs were hypothesized to serve as a promising cell line for investigating the pathogenesis of neural differentiation disorders in the PNS caused by congenital HCMV infection. In this work, SHEDs were demonstrated to be fully permissive to HCMV infection and the virus was able to complete its life cycle in SHEDs. Under neurogenic inductive conditions, HCMV infection of SHEDs caused an abnormal neural morphology. The expression of stem/neural cell markers was also disturbed by HCMV infection. The impairment of neural differentiation was mainly due to a reduction of intracellular cholesterol levels caused by HCMV infection. Sterol regulatory element binding protein-2 (SREBP2) is a critical transcription regulator that guides cholesterol synthesis. HCMV infection was shown to hinder the migration of SREBP2 into nucleus and resulted in perinuclear aggregations of SREBP2 during neural differentiation. Our findings provide new insights into the prevention and treatment of nervous system diseases caused by congenital HCMV infection.

**Supplementary Information:**

The online version contains supplementary material available at 10.1007/s00018-024-05278-0.

## Introduction

Human cytomegalovirus (HCMV), a member of the herpes virus family, is a significant contributor to congenital developmental birth defects. Its primary manifestations are observed in the nervous system [1, 2]. Approximately 10–15% of neonates with congenital HCMV infection exhibit a wide range of central nervous system (CNS) and/or peripheral nervous system (PNS) disorders. CNS disorders caused by congenital HCMV infection present as microcephaly, intracranial calcification, ventriculomegaly, among others. Concurrently, the main defects in PNS caused by HCMV infection include hearing loss, stemming from the absence of peripheral nerves or the loss of corresponding function. Furthermore, around 5–10% of infants congenitally infected with HCMV may not display symptoms at birth but later develop mild forms of brain disorders, including hearing loss and mental retardation [3]. However, the complete pathogenesis of neural differentiation disorders caused by HCMV infection remains to be fully elucidated.

Researchers have endeavored to clarify the mechanism behind neural differentiation disorders caused by HCMV infection. Odeberg et al. discovered that HCMV infection inhibits the differentiation of human neural precursor cells (NPCs) into neurons and astrocytes through apoptosis induced by virus in cells, during differentiation process [4, 5]. Additionally, certain studies suggest that HCMV may stimulate neuroinflammatory processes [6–8] and impede neural precursor cell proliferation by disrupting cell cycle control [4, 9]. Furthermore, Liu et al. reported that the HCMV immediate-early 1 protein (IE1) suppresses neural progenitor cell proliferation and neurosphere formation by downregulating the expression of hairy and enhancer of split 1 protein in infected NPCs [10, 11].

Several neural cell lines have been successfully isolated and cultured in vitro for research on human neural differentiation and development, such as neural stem cells (NSCs), NPCs and induced pluripotent stem cells (iPSCs) [12–14]. These cell lines also have been used to investigate the pathogenesis of congenital HCMV infection in neural differentiation disorders. However, it has been demonstrated that HCMV cannot complete a full viral replication in iPSCs [15]. Although NPCs are susceptible to HCMV infection, they represent an intermediate stage in the differentiation process from NSCs to mature neurons. Consequently, alterations in NPC differentiation caused by HCMV infection may not fully capture the overall impact of HCMV on stem cell differentiation [4, 5, 9, 16]. NSCs derived from the forebrain tissue of aborted human fetuses are considered an optimal model for studying neural differentiation post HCMV infection [15, 17]. Nevertheless, due to ethical considerations and limited availability, NSCs have not been extensively employed in this type of research. Considering the challenges associated with the use of NSCs, cell lines possessing both neural differentiation capacity and permissibility to HCMV infection would significantly facilitate further research in this field.

Stem cells from human exfoliated deciduous teeth (SHEDs), isolated by Miura et al. in 2003, present a novel population of MSCs derived from the neural crest of the ectoderm [18–20]. Demonstrating a high proliferation rate and multi-differentiation capacity, under various inductive conditions, SHEDs can successfully differentiate into diverse cell types, including neural cells, osteoblasts, hepatocytes, and pancreatic cells. Among them, SHED-derived neural cells have been used to investigate the occurrence and treatment of multiple nervous system diseases, such as Parkinson’s disease, acute contused spinal cord injury, and sciatic nerve enervation [21–23]. Since SHEDs originate from the neural crest of the early embryonic ectoderm, their differentiation process into neural cells more accurately reflects the differentiation and development of the PNS. Therefore, SHEDs were hypothesized to serve as a promising cell line for investigating the pathogenesis of neural differentiation disorders in the PNS caused by congenital HCMV infection.

The aim of this study was to investigate the molecular mechanism of neural differentiation disorders caused by HCMV infection in SHEDs. Initially, the susceptibility of SHEDs to HCMV was established. Genomic replication and mature particle packaging in SHEDs were assessed. Subsequently, under neurogenic inductive conditions, the neural differentiation of SHEDs following HCMV infection was characterized. The study also determined the altered expression of stem/neural cell markers caused by HCMV infection in SHEDs. Ultimately, the impairment of neural differentiation resulting from HCMV infection was found related to a reduction of sterol regulatory element binding protein 2 (SREBP2)-mediated cholesterol biosynthesis. The results of this study offer new insight into the prevention and treatment of nervous system diseases caused by congenital HCMV infection.

## Methods and materials

### Primary culture of SHED

Human exfoliated deciduous teeth of children were obtained as discarded biological samples collected from children aged 7- to 8-year-old from Department of Pediatric Dentistry of China Medical University. Isolation and characterization of SHED was performed as described previously [19]. To exclude the impact of individual genetic background on our findings, SHED cell lines used in this study were obtained from 10 children respectively. Experiments were conducted from multiple cell lines. Only 4 to 8 generations of SHEDs can be used for experiments and differentiation induction.

### Cell preparation

HELFs were cultured in Dulbecco’s minimal essential medium (DMEM, BI, Israel) containing 10% fetal bovine serum (Equitech, USA) and 100U/mL penicillin-streptomycin (BI, Israel) solution. SHEDs were cultured in α-MEM (Gibco, USA) containing 15% FBS (Equitech, USA) and 100U/ml penicillin-streptomycin (BI, Israel) solution.

Trypsin-EDTA (0.25% trypsin, 0.02% EDTA; Gbico, USA) was used for digestion during cell passage. Both HELF and SHEDs were maintained at 37 °C with 5% CO_2_ and passaged every 3–4 days at 90% confluence.

### Virus preparation

Human cytomegalovirus clinical low-passage strain Han and laboratory strain Towne expressing a GFP replorter under the SV40 promoter were derived from BAC DNA as previously described [24]. Towne is a present of Professor Yongjun Yu (Department of Cancer Biology, Abramson Family Cancer Research Institute, University of Pennsylvania, USA). Viral stocks were generated following viral propagation in HELFs by standard ultracentrifugation procedures described previously [25]. The purified virus particles were stored at -80 °C until use.

### Differentiation of SHEDs into neural cells

SHEDs were induced to differentiate into neural cells as previously described [18–20, 26–28]. Briefly, SHEDs were seeded (5000 cells/cm^2^) on 0.1% Gelatin (STEMCELL, Canada)-coated circular glass cover slips (20 mm in diameter; nest, China) in plates. The attached cells were induced differentiation (1mL/well) in Neurobasal A medium (Gibco, USA) containing 1×B27 (Invitrogen, USA) supplement, 40ng/mL human recombinant basic fibroblast growth factor (bFGF, STEMCELL, Canada), 20ng/mL human recombinant epidermal growth factor (EGF, STEMCELL, Canada) and 100U/mL penicillin-streptomycin. Fresh medium was changed every 24 h. Cells were allowed to differentiate for 7 days or extended to 14 days at 37 °C in an atmosphere of 5% CO_2_ and 95% O_2_ prior to fixation and the performance of IFA or other experiments.

### Detection of HCMV mRNAs and proteins

To detect viral molecules including HCMV mRNAs and proteins, we infected SHEDs or HELFs with higher multiplicities of infection (MOI) to make the detection of viral molecules better. SHEDs or HELFs were infected by HCMV at MOI of 1, and harvested at 24, 48 and 72 h post infection (hpi). Total RNA was extracted and reverse transcribed using RNeasy Mini Kit (QIAGEN, Germany) and QuantiNova Reverse Transcription Kit (QIAGEN, Germany) according to the supplier’s recommendations. Quantitative PCR was performed for transcripts of HCMV UL123, UL32, UL55, UL44, UL83, UL99 and glyceraldehyde 3-phosphate dehydrogenase (GAPDH) using QuantiNova SYBR Green Kit (GIAGEN, Germany). Information of primers used in this study was listed in Table S1. Three independent experimental replicates were performed, and the results are presented as means and standard deviation.

HCMV-infected SHEDs were harvested at 24, 48 and 72 hpi by being rinsed PBS, trypsinized, and pelleted. Total cellular protein was extracted by adding an equal volume of M-PER™ Mammalian Protein Extraction Reagent (ThermoFisher, USA) containing protease and phosphatase inhibitor cocktail (ThermoFisher, USA) to cell pellets. HCMV proteins were detected using western blotting with following primary antibodies (Abs): IE2/3 (anti-rabbit, abcam, ab37601), HCMV-gB (anti-mouse, Santacruz, sc-69,742), CMV-pp52 (anti-mouse, Santacruz, sc-69,744), CMV-pp28 (anti-mouse, Santacruz, sc-56,975) and GAPDH (anti-mouse, proteintech, 60004-1-lg). Blots were probed with secondary Abs conjugated with horseradish peroxidase (HRP) and visualized with enhanced chemiluminescence reagents (Millipore, USA). Each experiment was performed in triplicates with representative images shown.

### Quantification of HCMV DNA copies in supernatants

SHEDs were infected with HCMV at MOI of 1. Supernatants were collected at 24, 48, 72 and 96 hpi. The DNA copy numbers of HCMV genome in the supernatants were measured using CMV DNA quantification Kit (LifeRiver, China) on QuantStudio Q5 instrument (ThermoFisher, USA). Three independent experimental replicates were set at each time point and the results are presented as means and standard deviation.

### Immunofluorescence assay (IFA)

Cells with different treatments including differentiated SHEDs with HCMV infection were first fixed with buffer 4% paraformaldehyde for 30 min at room temperature and blocked with blocking solution (1×PBS containing 0.1% Triton X-100, 0.3 M glycine and 5% BSA) for 1 h. To avoid unwanted morphological changes caused by the overwhelming release of inflammatory cytokines, we infected differentiated SHEDs with a lower MOI of 0.5. Fc receptor blocking solution (absin, abs9476) was added to reduce nonspecific binding prior to incubation with primary antibodies for overnight at 4 °C, respectively. The samples were subsequently washed and incubated with secondary antibodies. Cells were counterstained with DAPI before mounting.

The following antibodies were used for IFA: Nestin (anti-mouse, cell signaling technology, 33475s), β3-tubulin (anti-mouse, cell signaling technology, 5568), MAP2 (anti-mouse, cell signaling technology, 4542), NeuN (anti-mouse, abcam, ab177487), SREBP1 (anti-rabbit, proteintech 14088-1-AP), SREBP2 (anti-rabbit, abcam, ab30682), SCAP (anti-mouse, abcam, ab190103), β-actin (anti-rabbit, abcam, ab179467), Alexa Fluor® 594-labeled Goat Anti-Rabbit IgG (H + L) (ZSGB-BIO, ZF-0516) and Alexa Fluor® 594-labeled Goat Anti-mouse IgG (H + L) (ZSGB-BIO, ZF-0513).

A Carl Zeiss LSM880 confocal microscope with NIS Elements was used for image acquisition and analysis. All of the experiments were performed in triplicates, and representative images were shown. The magnification for all images presented is 400×. Relative fluorescence values of cells in the field of vision were measured and the mean fluorescence value were calculated by Image J software. Mean fluorescence value = Total fluorescence value of the sight/Area of the sight.

### Western blots analysis

Cells with different treatments were harvested at indicated time points. An equal volume of M-PER™ Mammalian Protein Extraction Reagent (ThermoFisher, USA) containing protease and phosphatase inhibitor cocktail (ThermoFisher, USA) was added into the cell pellets. Nuclear extracts were prepared using NE-PER™ (ThermoFisher, USA) and cytoplasmic extraction reagents (ThermoFisher, USA) according to the manufacturer’s directions. Protein concentration was measured using BCA protein assay kit (Takara, Japan), equivalent amounts of denatured cell lysate were electrophoresed using SDS-polyacrylamide gel electrophoresis (SDS-PAGE) and transferred to PVDF membrane (Millipore, USA). And then the blots were probed with each of the following primary antibodies (Abs): nestin (anti-mouse, cell signaling technology, 33475s), β3-tubulin (anti-mouse, cell signaling technology, 5568), MAP2 (anti-mouse, cell signaling technology, 4542), NeuN (anti-mouse, abcam ab177487), IE2/3 (anti-rabbit, abcam, ab37601), SREBP2 (anti-rabbit, abcam 30,682), HMGCR (anti-rabbit, affinity DF6518), β-actin (anti-mouse, proteintech, 66009-1-lg) and Histone-H3 (anti-rabbit, proteintech, 17168-1-AP). After extensive washing, blots were probed with secondary Abs conjugated with horseradish peroxidase (HRP) to detect bound primary Abs. Proteins were visualized with enhanced chemiluminescence reagents, Immobilon Western HRP Substrate (Millipore, USA). All Western blot experiments were performed in triplicates, with representative images shown.

### Transcriptomic sequencing

Cell samples including SHEDs, differentiated SHEDs (SHEDi) and differentiated SHEDs (hSHEDi) with HCMV infection (MOI = 1) were harvested and lysed in TRIzol Reagent (Thermo Fisher, USA) for high throughput RNA-sequencing. After RNA quantification and qualification was assessed, a total amount of 1 µg RNA per sample was used as input material for the RNA sample preparations. Sequencing libraries were generated using NEBNext® UltraTM RNA Library Prep Kit for Illumina® (NEB, USA) following manufacturer’s recommendations and index codes were added to attribute sequences to each sample. Prepared libraries were sequenced on an Illumina Novaseq platform and 150 bp paired-end reads were generated.

Index of the reference genome was built and paired-end clean reads were aligned to the reference genome using Hisat2 v2.0.5. Differential expression analysis of two groups (three biological replicates per condition) was performed using the DESeq2 R package (1.16.1). The resulting *P*-values were adjusted using the Benjamini and Hochberg’s approach for controlling the false discovery rate. Genes with Fold Change ≥ 2 and an adjusted *P*-value < 0.05 found were assigned as differentially expressed.

Differentially expressed genes were functionally categorized and analyzed using Ingenuity Pathway Analysis software (QIAGEN Ingenuity System) and Gene Set Enrichment Analysis (GSEA, http://www.broadlinstitute.org/gsea).

### Chemical treatment

U18666A (MCE, HY-107433), a cholesterol synthesis inhibitor [29–31], was added to cells at a final concentration of 2 µg/ml at the initiation of differentiation. Fatostatin (MCE, HY-14452), a inhibitor of SREBP activation [32–36], was added to cells at a final concentration of 1µM at the initiation of differentiation. Cholesterol (MCE, HY-N0322) was added into the medium at a final concentration of 10 µg/ml at the initiation of differentiation and supplied to set concentration with the change of medium.

### Cholesterol assay

Cells with different treatment were harvested and lysed in M-PER™ Mammalian Protein Extraction Reagent (ThermoFisher, USA) containing protease and phosphatase inhibitor cocktail (ThermoFisher, USA). The samples were diluted with component E (applied) according to the appropriate concentration, and then tested for cholesterol assay including cellular total cholesterol, free cholesterol and cholesterol easter using Amplex® Red Cholesterol Assay Kit (ThermoFisher, USA) according to the manual instruction as previously described [37]. Each assessment was performed in triplicate, and the differences between samples were balanced by protein concentration.

### Brdu staining

SHEDs (5 × 10^3^/well) were seeded on glass coverlips and cultured for 2–3 days. The cultures were incubated with BrdU solution (1:100) (Invitrogen) for 20 h, and stained with a BrdU staining kit (Invitrogen) according to the manufacturer’s instructions. The samples were then stained with hematoxylin. BrdU positive and total cell numbers were counted in 10 images per subject. The number of BrdU-positive cells was indicated as a percentage of the total cell number. The BrdU assay was repeated on three independent samples for each experimental group.

### Quantitative RT-PCR

Total RNA was extracted by using RNeasy Mini Kit (QIAGEN, Germany) with on column DNase treatment. cDNA was generated from 1 µg total RNA using QuantiNova Reverse Transcription Kit (QIAGEN, Germany) according to the supplier’s recommendations. Quantitative PCR (qPCR) was performed on QuantStudio Q5 instrument (ThermoFisher, USA). Each 20 µl qPCR mixture contained 100ng reverse transcription product, 10 µl 2×QuantiNova SYBR Green PCR Master Mix, 2 µl QN ROX Reference Dye (QIAGEN, Germany), and 0.7µM forward (F) and reverse (R) primers. Primers used in this study were listed in Table S1. Amplification was performed by denaturation at 95 °C for 2 min, followed by 40 two-step cycles of 95 °C for 15s and 60 °C for 30s. Each reaction was performed in triplicates, and the results for the target gene mRNA were normalized to GAPDH using the 2^ΔΔCT^ method. The results are presented as means and standard deviation.

### Confocal microscopy

For the co-localization of SREBP and CMV UL44, SHEDs were seeded in cover glass-bottomed 24-well plates and fixed with buffer 4% paraformaldehyde for 30 min at room temperature and blocked with blocking solution (1×PBS containing 0.1% Triton X-100, 0.3 M glycine and 5% BSA) for 1 h. Primary antibodies (SREBP1, Proteintech 14088-1-AP; UL44, Santa Cruz, sc-56,971) were incubated with slides at 4 °C overnight. The samples were then washed and incubated with secondary antibodies.

Images were acquired using a Carl Zeiss LSM880 confocal microscope with NIS Elements. Image J were used for image analysis. All experiments were performed at least in triplicates, and representative images are shown. The magnification for all images presented is 400×.

### Statistical analysis

Statistical analyses were performed using Excel and GraphPad Prism 5.0. Data were expressed as mean ± SD. Statistically significant differences were evaluated using unpaired 2-tailed Student’s t test. In all cases, *P*-value of ≤ 0.05 was considered statistically significant.

## Results

### SHEDs are fully permissive to HCMV infection

**Fig. 1:**
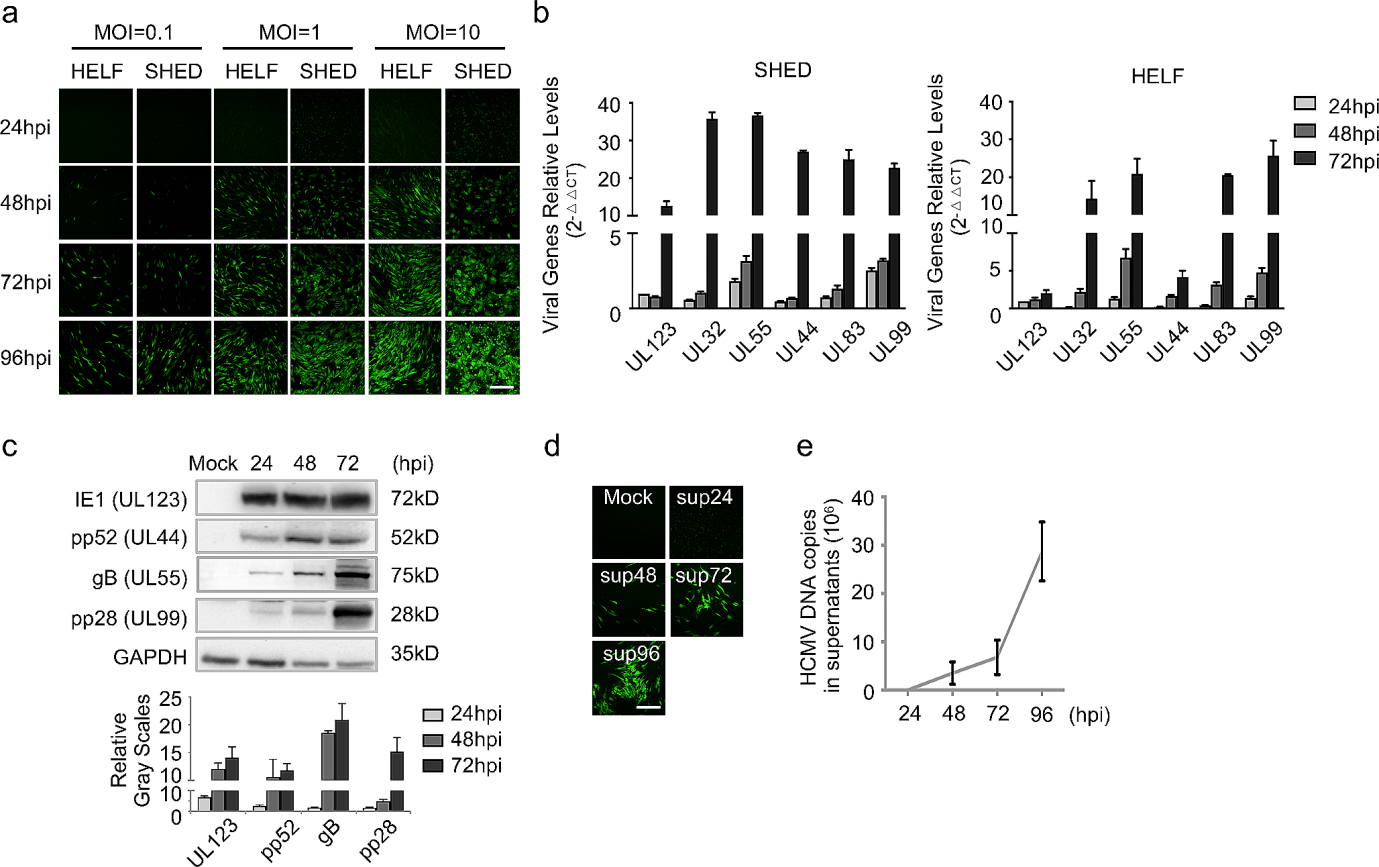
SHEDs are fully permissive to HCMV infection. **a** Increasing cell amounts with GFP expressed in HCMV-infected SHED (MOI = 0.1, 1 and10, respectively) and HELFs. Scale bar = 100 μm. Magnification of images is 200×. **b** Quantitative PCR analysis of essential viral genes in HCMV-infected SHEDs (MOI = 1) and HELFs at different time points. Three independent experimental replicates in each group; error bars: mean ± SD. **c** Western blot analysis of HCMV proteins (UL123, UL44, UL55 and UL99) in infected SHEDs at different time points (MOI = 1), three independent experimental replicates in each group; error bars: mean ± SD. **d** Infected SHEDs could release mature infectious HCMV particles into supernatants. HELFs infected through incubation with supernatants from HCMV-infected SHEDs, scale bar = 100 μm. Magnification of image is 200×. **e** Quantitative results of viral DNA copies in supernatants from HCMV-infected SHEDs at different time points, three independent experimental replicates in each group; error bars: mean ± SD

Prior to delving into the impact of HCMV infection on neural differentiation in SHEDs, the susceptibility of SHEDs to HCMV infection was assessed by infecting them with green fluorescent protein (GFP)-labeled clinical HCMV strain Han and laboratory strain Towne, at varying multiplicities of infection. The fluorescence of GFP was observed at different time points post-infection. GFP fluorescence was detectable as early as 24 h post infection (hpi), indicating successful HCMV entry into SHEDs (Fig. 1a and Supplementary Fig. S1a). Since neural differentiation is a long-term process, assessments of viral replications of Han and Towne in SHEDs were extended to 14 days at a low dose (MOI = 0.1). The GFP levels continued to rise over the course of infection, ultimately leading to lytic infection (Supplementary Fig. S1b).

Next, the transcription of essential viral genes representing different viral phases was measured at multiple time points. Examined genes included UL123 (encoding IE1, immediate early phase), UL32/UL44/UL55 (early phases), and UL83/UL99 (late phases). Similar to findings in HCMV-infected human embryonic lung fibroblasts (HELFs), transcription of these viral genes was confirmed in SHEDs, with gene accumulation gradually increasing during infection (Fig. 1b). Notably, the transcription of UL123 and UL44 appeared stronger in SHEDs compared to HELFs. Furthermore, the expression of viral proteins (IE1, UL44, UL55, and UL99) was also detected, with the kinetics of viral protein expressions aligning with their transcription levels (Fig. 1c).

Subsequent assessment focused on whether mature HCMV particles were produced in SHEDs during infection. Supernatants from HCMV-infected SHEDs were collected at 24, 48, 72 and 96 hpi, respectively, and incubated with HELFs. After 5 days of exposure to the supernatants from infected SHEDs, cell clusters with GFP fluorescence were detected and counted, indicating the release of mature infectious HCMV particles from SHEDs into the supernatants. No GFP was detected in HELFs incubated with supernatant from mock-infected or early-infected (24 hpi) SHEDs, confirming that the mature virus particles were assembled and released into the supernatants only during the late phase of infection (Fig. 1d). Further quantitative analysis demonstrated the accumulation of viral DNA in the supernatants throughout infection, revealing the continuous release of HCMV particles from SHEDs post infection (Fig. 1e).

Considering the neurogenic differentiation capacity of SHEDs and their full permissibility to HCMV infection, it was concluded that SHEDs can serve as a promising cell line for investigating the mechanism of neural differentiation disorders caused by HCMV infection in vitro.

### HCMV infection impairs neural differentiation of SHEDs

To investigate the impact of HCMV infection on neural differentiation, SHEDs were induced to differentiate into neural cells with or without HCMV infection. Throughout the neurogenic differentiation process, uninfected SHEDs exhibited morphological changes from their original stem cell structure characterized by a high nuclear-cytoplasmic ratio resembling bird nests without distinct boundaries to a neuron-like structure. This transformation included cell elongation, axonal structure formation, and a more abundant cytoplasm. In contrast, HCMV-infected SHEDs showed an abnormal neural morphology, showcasing reduced cell volume due to cytoplasm reduction and the absence of axon formation under neurogenic inductive conditions (Supplementary Fig. S2a and S2b).

To assess the neural differentiation process, molecular markers of both stem cells and neural cells were detected, including Nestin, β3-tubulin, microtubule-associated protein 2 (MAP2) and neural nuclei (NeuN). Nestin is abundantly expressed in NSCs and NPCs [38–40], while β3-tubulin, MAP2, and NeuN are expressed in mature neural cells [41–43]. β3-tubulin and MAP2 serve as cytoskeletal proteins involved in neural cell structure formation, mainly distributed in the cytoplasm, and NeuN localized in the nucleus of mature neural cells.

**Fig. 2:**
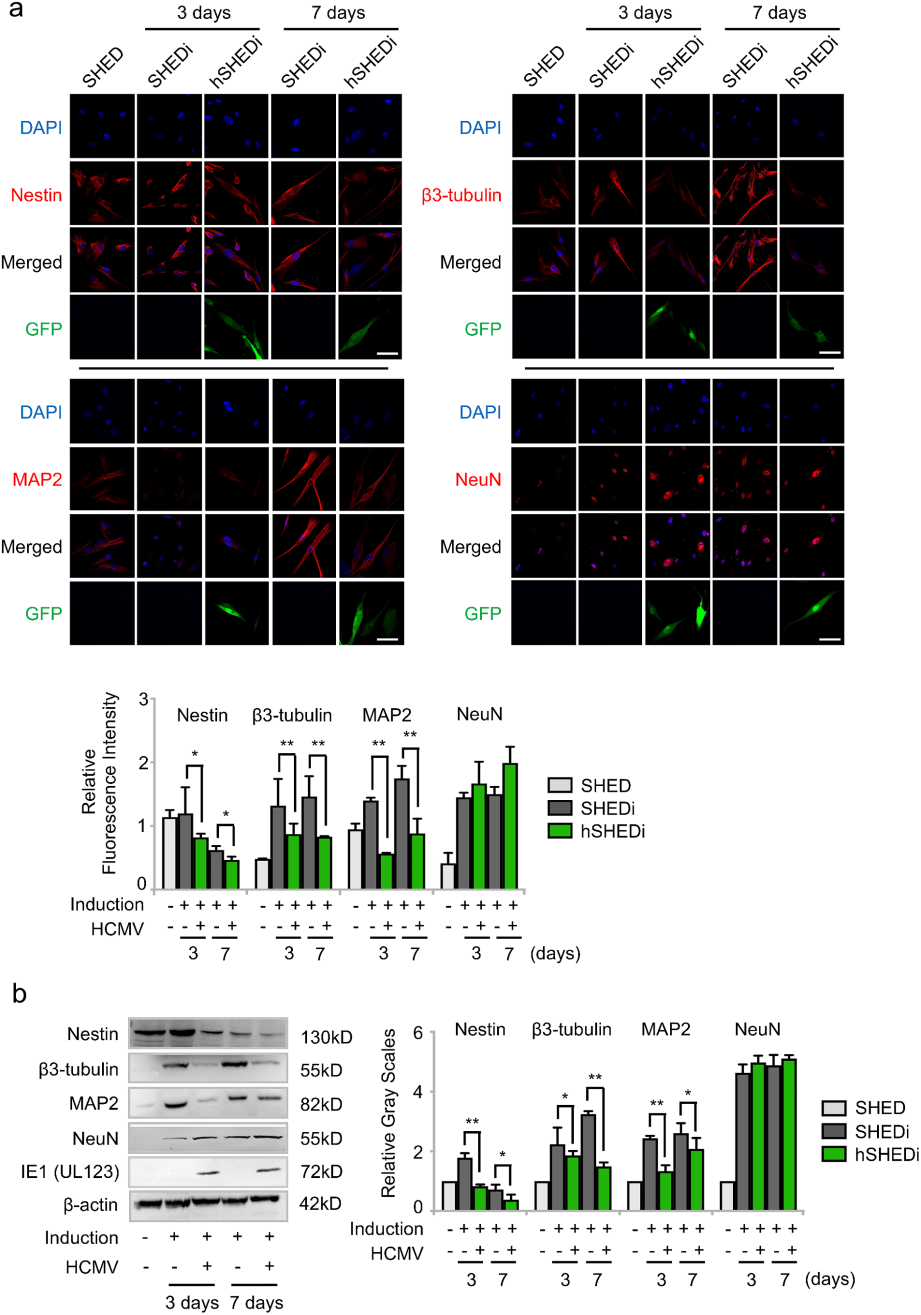
HCMV infection impairs neural differentiation. **a** SHEDs with or without HCMV infection, stained for molecular markers (Red), including Nestin, β3-tubulin, microtubule-associated protein 2 (MAP2) and neural nuclei (NeuN) at 3 and 7 days under neurogenic inductive conditions. Nuclei were counterstained with DAPI (blue). SHEDi: SHEDs under neurogenic inductive conditions; hSHEDi: SHEDi infected with HCMV clinical strain Han at an MOI of 0.5; scale bar = 50 μm. Magnification of image is 400×. Relative fluorescence values were measured using Image J software, three independent experimental replicates in each group; bar graphs for SHEDi were in gray color; bar graphs for hSHEDi were in green color; error bars: mean ± SD; *P < 0.05; **P < 0.01. **b** Western blot analysis of Nestin, β3-tubulin, MAP2 and NeuN in SHEDs during differentiation. SHEDi: SHEDs under neurogenic inductive conditions; hSHEDi: SHEDi infected with HCMV clinical strain Han at an MOI of 0.5. The relative gray values were measured by Image J software, three independent experimental replicates in each group; bar graphs for SHEDi were in gray color; bar graphs for hSHEDi were in green color; error bars: mean ± SD; *P < 0.05; **P < 0.01

Compared to undifferentiated SHEDs, the expression of Nestin was significantly decreased by 37.80 ± 9.80% in SHEDs at 7 days of differentiation, indicating the loss of differentiation properties of stem cells. On the contrary, the expressions of β3-tubulin, MAP2 and NeuN were gradually increased in SHEDs from 3 days of differentiation, demonstrating gains of mature neural properties under neurogenic inductive conditions (Fig. 2a).

In HCMV-infected SHEDs, compared to those with stem cell properties, Nestin expression decreased by approximately 29.19 ± 3.56% at 3 days of differentiation and by 59.37 ± 7.21% at 7 days of differentiation. Additionally, compared to SHEDs with induction, the expressions of β3-tubulin and MAP2 were decreased by 49.50 ± 8.56% and 46.28 ± 3.80% at 7 days of differentiation respectively, indicating a lack of substantial increase in β3-tubulin or MAP2 expression under neurogenic inductive conditions. In addition, there was no significant difference of NeuN expression between differentiated SHEDs with or without HCMV infection. Moreover, no variance in the cellular distribution of these molecular markers was observed among the different groups (Fig. 2a). Subsequently, changes in the expression of these molecular markers were confirmed by western blot analysis (Fig. 2b). Taken together, these findings suggest that HCMV infection impairs the differentiation of SHEDs into neural cells.

### HCMV infection reduces intracellular cholesterol levels during neural differentiation

**Fig. 3:**
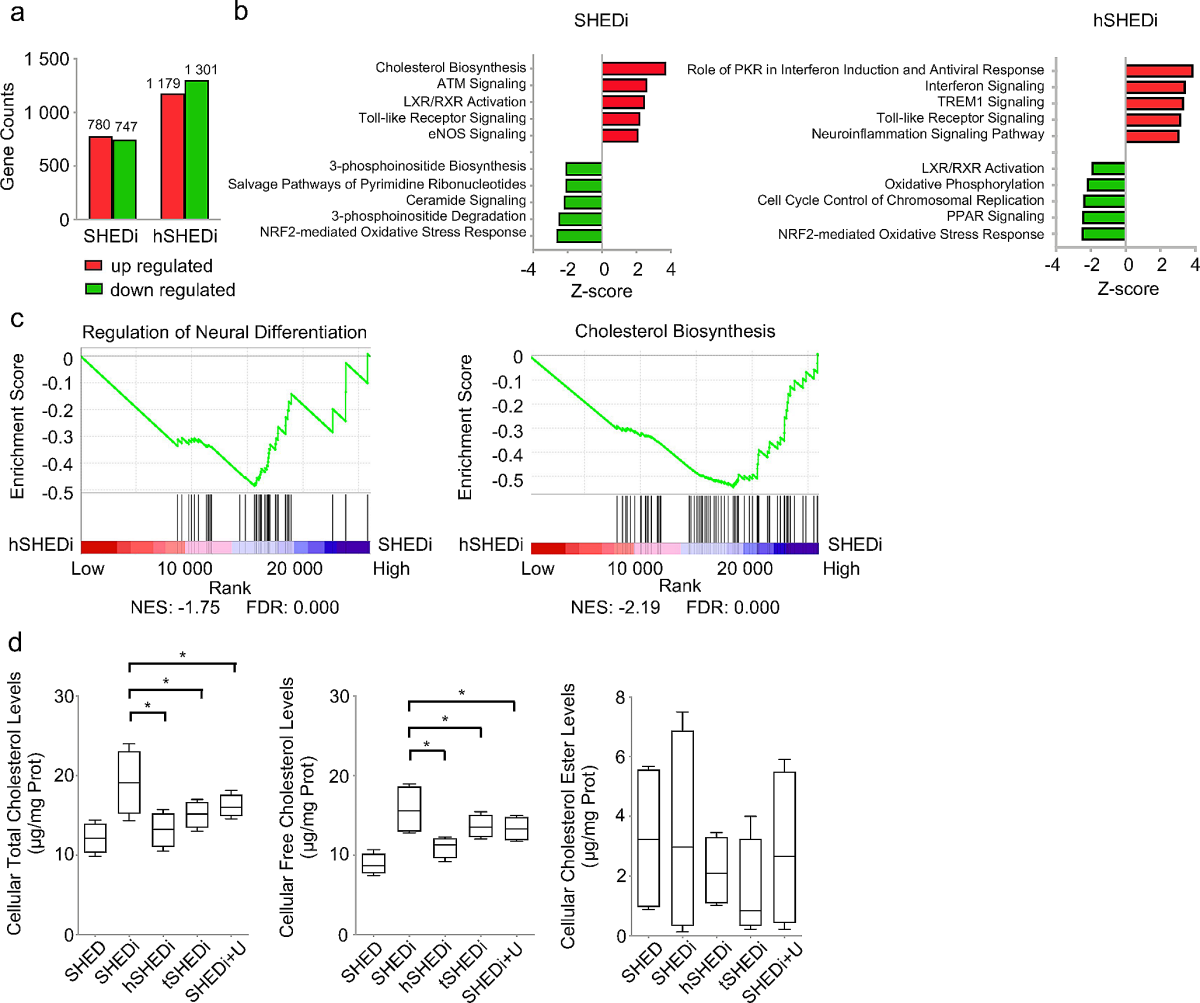
HCMV infection reduces intracellular cholesterol biosynthesis during neural differentiation. **a** RNA-sequencing data analysis of transcriptomic profiles of SHEDs altered by HCMV infection during differentiation. **b** Differentially expressed genes (DEGs) involved pathways analyzed using ingenuity pathway analysis (IPA) software. **c** The inhibition in neural differentiation pathway (left) and cholesterol biosynthesis pathway (right) confirmed by GSEA analysis. **d** Intracellular cholesterol contents measured in SHEDs with different treatment. Cells added U18666A, an inhibitor specific to cholesterol synthesis, were measured as positive reference. SHEDi: SHEDs under neurogenic inductive conditions; hSHEDi: SHEDi with HCMV clinical strain Han at an MOI of 0.5; tSHEDi: SHEDi with HCMV laboratory strain Towne at an MOI of 0.5. Triple independent experiments in each group; error bars: mean ± SD; *P < 0.05

To investigate the mechanism behind neural differentiation impairment caused by HCMV infection, RNA-sequencing analysis in differentiated SHEDs was performed, with or without HCMV infection. Cells were harvested at the 3 days of differentiation, a time point marked by observable changes in neural differentiation-associated markers and completion of a round of viral genomic replication and transcription. Compared to SHEDs without induction, the transcriptions of 780 genes were increased and the transcriptions of 747 genes were decreased at the 7 days of differentiation (Fig. 3a). In differentiated SHEDs with HCMV infection, the transcription of 1,179 genes increased, while 1,301 genes decreased. Notably, 470 upregulated genes and 572 downregulated genes were shared between uninfected and HCMV-infected differentiated SHEDs, indicating distinct transcriptional profiles due to the notable impact of HCMV infection on transcriptional regulation during neural differentiation.

Pathway analysis utilizing Ingenuity Pathway Analysis (IPA) software revealed significant influences on pathways related to biosynthesis during the differentiation of SHEDs into neural cells. Specifically, cholesterol biosynthesis exhibited activation with a *z*-score of 3.74, while the pathway involving 3-phosphoinositide biosynthesis was suppressed with a *z*-score of -2.11. It was inferred that the neural differentiation of stem cells coincided with alterations in metabolic processes. In HCMV-infected cells, activation of pathways associated with the antiviral response, such as interferon and Toll-like receptor signaling pathways, was observed. However, the activation of cholesterol biosynthesis disappeared, suggesting that, apart from immune responses, HCMV infection might disrupt the cholesterol biosynthesis process during neural differentiation (Fig. 3b). It could be speculated that, except for immune responses caused by infection, HCMV infection potentially disturbs the cholesterol biosynthesis process during neural differentiation.

Gene set enrichment analysis (GSEA) was performed to further assess the effect of HCMV infection on neural differentiation and cholesterol biosynthesis, with normalized enrichment scores (NES) indicating the degree of gene enrichment among these pathways. NES values exceeding 1.60 or falling below − 1.60 signify significant enrichment of genes in the pathway. In our results, the NES values for neural differentiation and cholesterol biosynthesis pathways were − 1.75 and − 2.19, respectively (Fig. 3c), indicating suppression of both pathways by HCMV infection in SHEDs under neurogenic inductive conditions.

To validate the impacts of HCMV infection on cholesterol biosynthesis, intracellular cholesterol contents were measured in SHEDs with different treatments. U18666A, which suppresses the activities of desmosterol reductase, sterol–D8–D7 isomerase and oxidosqualene cyclase, was used as a cholesterol synthesis inhibitor in our study [29–31]. Induced SHEDs treated with U18666A were also included as positive control. As known, intracellular cholesterol primarily exists in two forms: free cholesterol (FC) and cholesterol ester (CE), with their total amounts combined to calculate the total cholesterol (TC) content. Our results showed that the intracellular cholesterol contents differed between uninfected and HCMV-infected differentiated SHEDs (Fig. 3d). Compared to SHEDs without induction, the TC and FC contents significantly increased in SHEDs at 7 days of neurogenic differentiation. In HCMV-infected SHEDs (Han or Towne), the contents of TC and FC decreased by approximately 50 ± 6.80% compared to uninfected cells, reaching levels equivalent to those in U18666A-treated cells. Importantly, there was no significant difference in cellular viabilities among cells subjected to different treatments (Supplementary Fig. S3a). The changes in TC content closely mirrored those in FC content. Furthermore, no significant difference in CE contents was observed among all groups (*P* = 0.1184). Additionally, FC and TC contents were measured in undifferentiated SHEDs with or without infection. It was observed that HCMV infection led to an increase in FC content by about 46 ± 3.98% in cells without induction, as previously described (Supplementary Fig. S3b). These findings suggest that the TC content increased to support the differentiation of SHEDs into neural cells, while HCMV infection hindered intracellular FC biosynthesis, resulting in a decrease in TC content during differentiation.

### Reduction of cholesterol contents causes impairment of neural differentiation

**Fig. 4:**
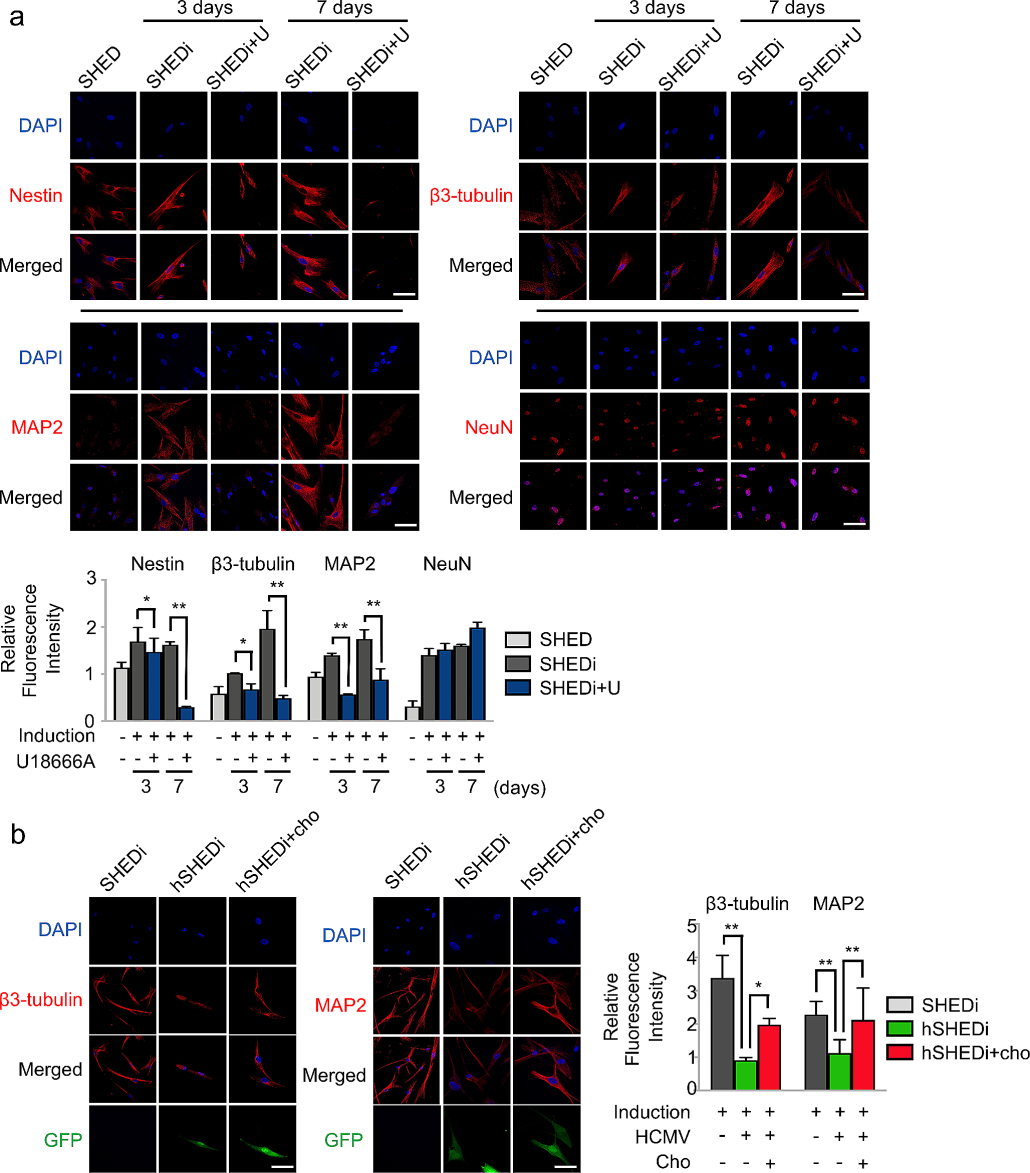
Reduction of cholesterol biosynthesis causes impairment of neural differentiation. **a** IFA for the indicated markers in SHEDs under neurogenic inductive conditions in the presence or absence of 2 µg/ml U18666A. SHEDi, SHEDs under neurogenic inductive conditions; SHEDi + U, SHEDi with U18666A added. Bar graphs for SHEDi were in gray color; bar graphs for SHEDi + U were in blue color; scale bar = 50 μm. Magnification of image is 400×. Relative fluorescence values were measured using ImageJ software, triple independent experiments in each group; error bars: mean ± SD; *P < 0.05; **P < 0.01. **b** IFA for β3-tubulin and MAP2 in infected SHEDs under neurogenic inductive conditions in the presence or absence of 10 µg/ml cholesterol. SHEDi, SHEDs under neurogenic inductive conditions; hSHEDi, SHEDi with HCMV infectio at an MOI of 0.5n; hSHEDi + cho: hSHEDi with cholesterol added. scale bar = 50 μm. Magnification of image is 400×. Relative fluorescence values were measured using ImageJ software, triple independent experiments in each group; error bars: mean ± SD; *P < 0.05; **P < 0.01

To investigate whether the reduction of cholesterol biosynthesis due to HCMV infection is a plausible reason for the impairment of neural differentiation, markers related to neural differentiation were assessed in differentiated SHEDs treated with U18666A. The reduction in intracellular cholesterol levels disrupted the neurogenic differentiation of SHEDs, and the dysregulation of these markers mirrored that caused by HCMV infection. In SHEDs with inhibited cholesterol synthesis through U18666A treatment, Nestin levels dropped to 26.04 ± 7.12% at 7 days of neurogenic differentiation compared to untreated SHEDs. Similarly, the levels of β3-tubulin and MAP2 decreased at both 3 and 7 days of differentiation. However, no significant effect on NeuN expression was observed (Fig. 4a).

Moreover, the addition of exogenous cholesterol into the medium at a final concentration of 10 µg/mL under neural inductive conditions rescued the neural differentiation properties in HCMV-infected SHEDs. In comparison to cells without cholesterol treatment, the expression levels of β3-tubulin and MAP-2 increased by 54.04 ± 4.79% and 46.93 ± 8.84%, respectively, at 7 days of differentiation (Fig. 4b). These results underscore the link between neural differentiation and cholesterol production, emphasizing that the reduction in cholesterol synthesis caused by HCMV infection directly impairs neural differentiation. Therefore, the application of exogenous cholesterol proves beneficial in mitigating the detrimental effects of HCMV infection during neural differentiation.

### HCMV infection represses SREBPs-mediated transcription during neural differentiation

**Fig. 5:**
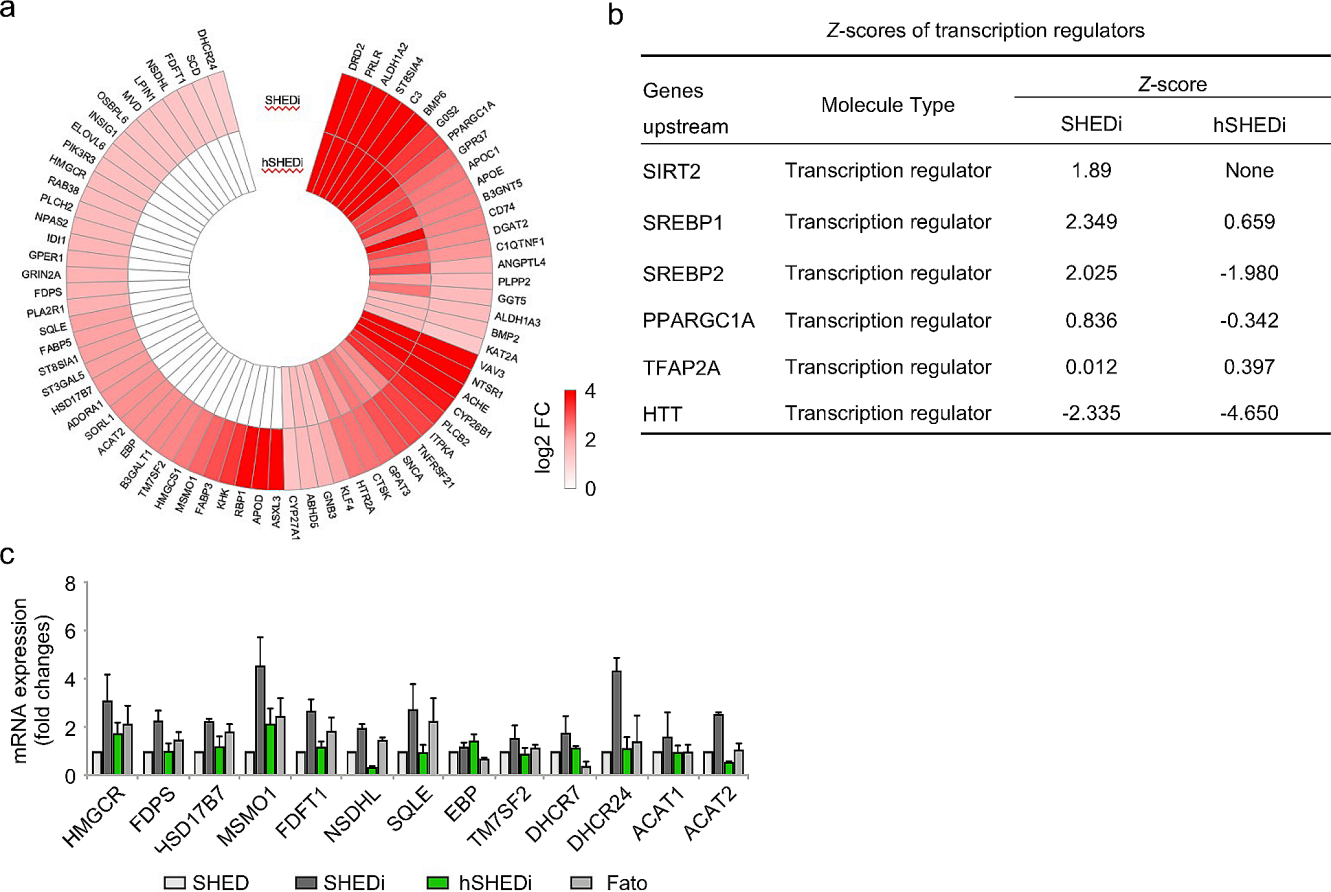
HCMV infection represses SREBPs-mediated transcriptions during neural differentiation. **a** The heat map indicating transcriptional inhibition of molecules involved in sterol biosynthesis by HCMV infection. **b** Activities of transcription regulators related to cholesterol biosynthesis evaluated and compared between SHEDs with or without HCMV infection (shown with *z*-score). SREBP1 and SREBP2 presented significant activations in induced SHEDs and opposite suppressions post HCMV infection. **c** qRT-PCR analysis of enzymes involved in cholesterol biosynthesis pathway at a transcriptional level by HCMV infection. Inhibitor of SREBPs, Fatostatin was added to induced SHEDs (1µM) as a reference control. SHEDi, SHEDs under neurogenic inductive conditions; hSHEDi, SHEDi with HCMV infection at an MOI of 1; Fato, SHEDi with Fatostatin added. Triple independent experiments in each group; error bars: mean ± SD

To investigate the mechanism under cholesterol biosynthesis reduction caused by HCMV infection, the genes involved in sterol biosynthesis were identified from the transcriptomic data and subjected to further analysis. The heat map reveals that 73 genes participating in sterol biosynthesis were upregulated during differentiation, with 23 of them identified as enzymes directly involved in cholesterol synthesis (Fig. 5a). In HCMV-infected cells, only 37 genes (50.68%, 37 vs. 73) were upregulated, indicating that HCMV infection inhibited the transcription of many genes. Detailed information is provided in Table S2. It is inferred that the reduction in cholesterol synthesis caused by HCMV infection may result from the inhibition of the transcription of most genes involved in cholesterol synthesis.

Building on these findings, the activities of transcriptional regulators associated with cholesterol synthesis were assessed by the *z*-scores of candidate transcriptional regulators and compared between different cell treatment groups (Fig. 5b). Among them, SREBPs, including SREBP1 and SREBP2, exhibited significant activations in differentiated SHEDs (*z*-score: 2.349 and 2.25, respectively) and inverse suppressions after HCMV infection (*z*-score: 0.659 and − 1.98, respectively). This data strongly supports the inhibition of SREBP function by HCMV infection.

SREBPs are transcriptional factors that maintain lipid homeostasis by controlling the expression of downstream enzymes involved in the biosynthesis of cholesterol and unsaturated fatty acids. Therefore, the investigation delved into whether the decreased expression of enzymes in the cholesterol biosynthetic pathway resulted from the inhibition of SREBPs by HCMV infection. Fatostatin, a chemical known to inhibit SREBP activities, was introduced into the culture medium of SHEDs undergoing induction [32–36]. Transcription levels of related enzymes were measured and compared among different cell treatment groups. The results indicated that genes inhibited by Fatostatin were also downregulated targets of HCMV infection during differentiation (Fig. 5c). HCMV infection had similar effects to Fatostatin on downstream genes of SREBPs. Among them, 3-hydroxy-3-methylglutaryl-coenzyme A reductase (HMGCR), generally considered a rate-limiting enzyme in the cholesterol biosynthetic pathway and a critical downstream molecule of SREBPs [44, 45], exhibited increased transcription during neural differentiation compared to normal SHEDs. However, it decreased with HCMV infection and Fatostatin treatment. These changes in HMGCR were also confirmed at the protein level (Supplementary Fig. S4). These findings suggest that HCMV might reduce intracellular cholesterol synthesis by inhibiting SREBPs-mediated transcription during neurogenic differentiation.

### HCMV infection hinders the migration of SREBP2 into nucleus

**Fig. 6:**
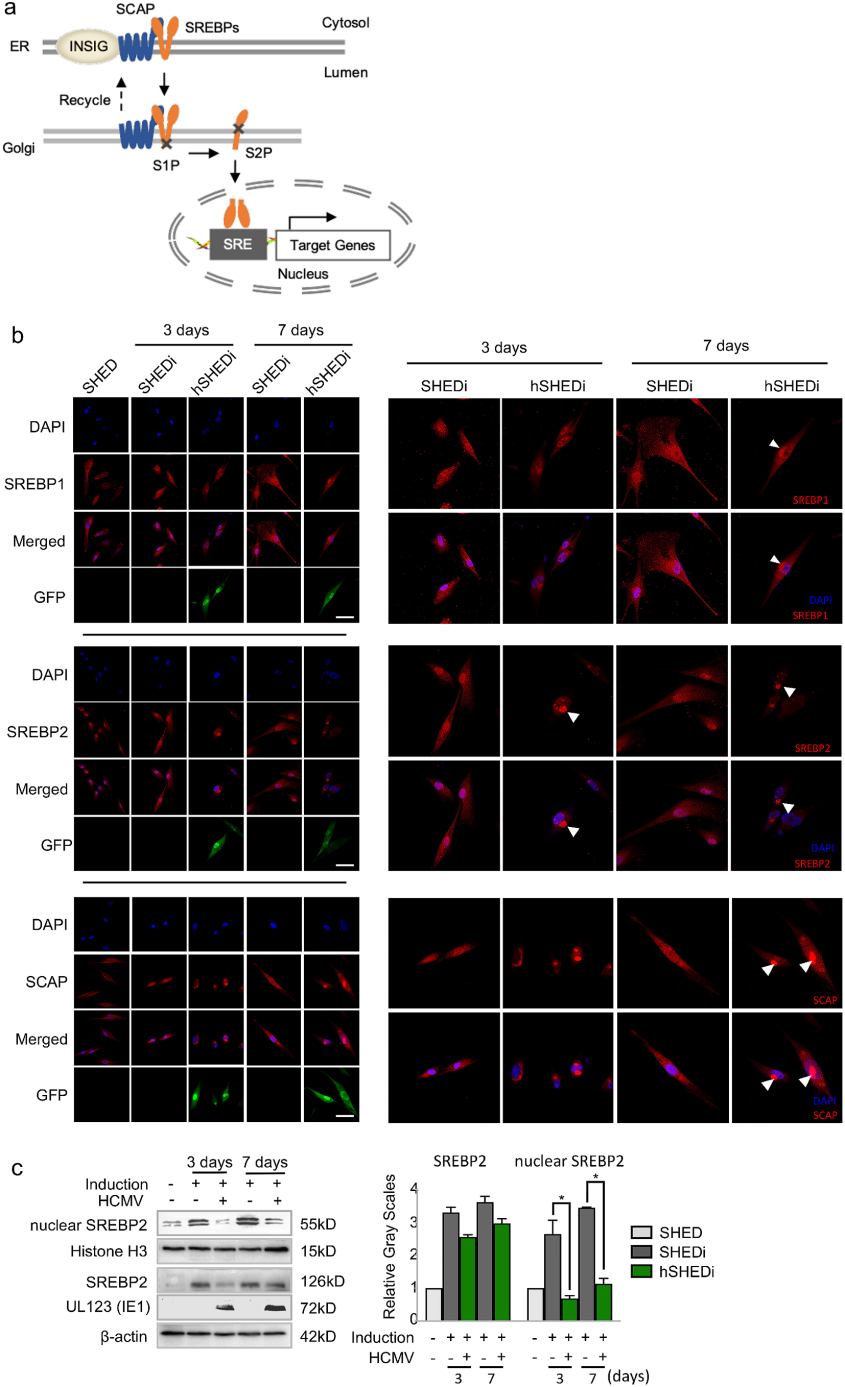
HCMV infection impedes the migration of SREBP2 into nucleus. **a** The schematic diagram of the production and translocation process mature SREBPs (see text for details). SREBP: sterol regulatory element binding protein; SCAP: SREBP cleavage-activation protein; INSIG: insulin-induced gene 1; SRE: sterol regulatory element; S1P: site-1 protease; S2P: site-2 protease; ER: endoplasmic reticulum. **b** IFA results for the intracellular localization of SREBP-SCAP components (red) in SHEDs under neural differentiation conditions with or without HCMV infection. Nuclei were counterstained with DAPI (blue). SHEDi, SHEDs under neurogenic inductive conditions; hSHEDi, SHEDi with HCMV infection at an MOI of 0.5; scale bar = 50 μm. Magnification of image is 400×. White triangles indicate perinuclear aggregation. **c** Western blots results showing a reduction of cytoplasm and nuclear SREBP2 caused by HCMV infection. SHEDi, SHEDs under neurogenic inductive conditions; hSHEDi, SHEDi with HCMV infection at an MOI of 0.5. The relative gray values were measured by Image J software, triple independent experiments in each group; bar graphs for SHEDi were in gray color; bar graphs for hSHEDi were in green color; error bars: mean ± SD; *P < 0.05

The generation of SREBPs with transcriptional regulatory activity involves the transport of the SREBP–SCAP complex from the endoplasmic reticulum (ER) to the Golgi apparatus. The inactive precursors of SREBPs (pre-SREBPs) anchor in the ER membranes in the complex with SREBP cleavage activation protein (SCAP). When endogenous cholesterol synthesis is required, the SREBP–SCAP complex is loaded into coat protein II (COP II) vesicles and transported to the Golgi apparatus. SREBPs are cleaved into nuclear SREBPs and then migrate into the nucleus, binding to sterol regulatory element (SRE) sequences in the promoter of several genes encoding enzymes essential for lipid synthesis and uptake (Fig. 6a).

SREBPs consist of SREBP1 and SREBP2. SREBP1 predominantly activates the transcription of genes required for fatty acid and triglyceride synthesis, while SREBP2 modulates transcription of genes necessary for cholesterol synthesis and uptake, such as HMG CoA synthase [46, 47]. Immunofluorescence assays (IFA) were employed to detect the cellular localizations of SREBP1 and SREBP2 in SHEDs separately to examine the impact of HCMV on them. In uninfected cells, the cytoplasmic and nuclear amounts of SREBP1 and SREBP2 increased with the progression of differentiation, indicating the activation of lipid synthesis, including fatty acid and cholesterol synthesis. Concurrently with the neural differentiation of SHEDs, more SREBP2 migrated into the nucleus, consistent with the increased TC content observed in our results (Fig. 6b).

Additionally, HCMV infection disrupted the cellular localization of SREBPs during neural differentiation (Fig. 6b). Centralized and noticeable aggregation of SREBPs around the nucleus was observed early at 3 days of differentiation in HCMV-infected cells. The migration of SREBP2 into the nucleus was impeded by HCMV infection, with the amounts of SREBP2 in the nucleus decreased by 56.66 ± 3.21% and 69.23 ± 4.01% at 3 and 7 days of differentiation, respectively, compared to uninfected cells. The SCAP protein was solely distributed in the cytoplasm, with no significant change in SCAP amounts observed among cells with different treatments. Perinuclear aggregation of SCAP was also noted along with SREBPs.

Moreover, multiple small foci stained by antibodies of SREBPs were found scattered within the nucleus. To determine whether the SREBP-formed small foci in the nucleus was due to the intensive binding of SREBPs to the viral genome, immunofluorescence co-localization analysis was performed for SREBPs and HCMV UL44 protein at 12 hpi [48]. Since HCMV UL44 mediates the replication of the viral genome, the intranuclear localization of UL44 can approximately represent the location of the viral genome. However, no co-localization was observed for SREBPs and HCMV UL44 protein (Supplementary Fig. S5).

To address whether HCMV infection could impede SREBP2 migration into nucleus, the amounts of SREBP2 in cytoplasm and nucleus from different groups were also measured using western blotting (Fig. 6c). The SREBP2 in cytoplasm and nucleus at the 3 and 7 days of differentiation were both decreased significantly after HCMV infection. In comparison to uninfected cells, the content of SREBP2 in the nucleus was reduced by 67.20 ± 4.21% and 73.86 ± 5.99% at 3 and 7 days of differentiation, respectively. These findings suggest that the impairment of neurogenic differentiation caused by HCMV infection potentially associated with a reduction of cholesterol contents, stemming from the hindered migration of SREBP2 into the nucleus.

## Discussion

The development and maturation of the nervous system is a gradual and complex process that involves the differentiation of neural tissue and the morphological differentiation of the encephalon. These processes encompass neurogenesis, migration, and the formation of axons and synapses. It has been demonstrated that cytomegalovirus can invade the nervous system at any stage of neurodevelopment, impeding the proliferation and differentiation of neural cells and causing neurodevelopmental disorders or other neurological diseases through acute or persistent viral infection [49].

Nervous system disorders caused by congenital HCMV infection include periventricular calcification, ventricular enlargement, microcephaly, mental retardation, congenital deafness, epilepsy, and autism [2]. However, until now, information about their pathogenesis has been limited.

The establishment of *in-vivo* and *in-vitro* models suitable for investigating the pathogenesis of HCMV infection in the nervous system is necessary. Animal models like murine cytomegalovirus infection model and guinea pig cytomegalovirus infection model, have been used to study the mechanism of cytomegalovirus disrupting cerebral development processes [50, 51]. However, due to the artificial routes of cytomegalovirus infection in most models and the limited sequence homology between human and animal cytomegalovirus genomes, the exact mechanism of HCMV infection causing human nervous system diseases cannot be fully elucidated using these animal models. Therefore, most studies on the mechanism of nervous system disorders or defects caused by congenital HCMV infection have predominantly been conducted in human-derived cells.

Researchers have attempted to use human iPSCs for this purpose, however, iPSCs have regrettably proven to be restrictive to HCMV infection [15]. Neural precursor cells and neural progenitor cells have been shown to support productive viral replication [9–11, 16, 52–54], and studies have demonstrated the contributions of IE1, IE2, and viral microRNAs to neuropathogenesis. Human NSCs, isolated from the forebrain tissue of an aborted fetus, are the most frequently used cell types in terms of cell sources, but their acquisition is challenging due to medical ethical concerns. Therefore, the SHED cell line serves as a convenient tool to study the mechanisms of neural differentiation or developmental disorders caused by congenital HCMV infection.

In SHEDs, HCMV was initially confirmed to complete viral entry, genomic transcription, replication, and the packaging and release of mature virus particles successfully. In addition, the transcription levels of critical viral genes in SHEDs and HELFs at different time points of infection were detected and compared. The transcriptional dynamics of most genes were very similar in HELFs and SHEDs. However, the transcription levels of UL123 and UL44 were higher in SHEDs than in HELFs at 72 hpi. HCMV UL123 is a key regulator initiating viral genomic transcription, and UL44 is an accessory protein of HCMV DNA polymerase, which is involved in the replication of the viral DNA genome [55, 56]. Therefore, the replication and transcription of the HCMV genome might be more permissive in SHEDs.

In our study, HCMV infection was demonstrated to impair the differentiation of SHEDs into neural cells. HCMV-infected SHEDs exhibited a shrunken cell volume due to the reduction of the cytoplasm and the lack of neural axon formation under neurogenic inductive conditions. In addition, the expression of Nestin was decreased by about 29.19 ± 3.56% at the 3 days of differentiation and even lower (59.37 ± 7.21%) at the 7 days of differentiation compared to that in noninduced cells, indicating a rapid loss of the differentiation properties of the stem cells. Consequently, no increase was observed for β3-tubulin or MAP2 post-induction. This result might be due to the loss of the differentiation capacity. Nestin, β3-tubulin and MAP2 are cytoskeletal proteins involved in the formation of cell structures. The reduced expression of these cytoskeletal proteins might lead to the abnormal morphology of SHEDs under neurogenic inductive conditions.

Some viruses have been identified to interfere with host cholesterol metabolism during infection. For instance, hepatitis C virus genes have been proven to alter host cell cholesterol/lipid metabolism by stimulating the phosphorylation of SREBPs and proteolytic processing of SREBP2, thus inducing hepatic steatosis [57, 58]. Similarly, HCMV has been reported to manipulate the cellular cholesterol efflux pathway to modify host plasma membrane properties for better virus dissemination during lytic infection [59, 60]. Activation of liver X receptor has been demonstrated to decrease the assembly of HCMV particles in foreskin fibroblasts by reducing cholesterol content [61]. In our study, we also measured the TC and FC levels in HCMV-infected SHEDs without induction. Consistent with previous findings, HCMV infection led to an elevation of cholesterol content in SHEDs. However, under neurogenic inductive conditions, HCMV infection caused a significant decrease in both FC and TC compared to SHEDs without infection. It is speculated that the suppression of cholesterol synthesis is a specific effect of HCMV infection during the differentiation process.

Cholesterol is abundant in the nervous system. During the development of the nervous system, cholesterol is involved in regulating the proliferation, synapse formation, astrocyte proliferation, nerve repair, and remodeling of NSCs and NPCs [62]. Cholesterol also serves as determinant in stabilizing the endogenous membrane structure, as well as nerve cell growth and differentiation [33, 63]. According to our results, the level of intracellular cholesterol increased during the differentiation of SHEDs into neural cells. In particular, the contents of FC and TC in SHEDs increased by 38.60% and 22.90%, respectively. However, this increase in cholesterol content was eliminated by HCMV infection. A simple reduction of intracellular cholesterol was confirmed to block neural differentiation. Therefore, it was speculated that HCMV could impair neural differentiation via reducing intracellular cholesterol production.

The synthesis of intracellular cholesterol is controlled by a series of enzyme activation reactions, in which the changes in the expression level and activities of various enzymes are the main regulatory factors. During the differentiation of SHEDs into neural cells, Most of the enzymes in the cholesterol synthesis pathway were significantly increased transcriptionally; while after HCMV infection, these levels were significantly decreased. Thus, HCMV infection potentially inhibits intracellular cholesterol synthesis transcriptionally.

SREBPs are crucial transcriptional regulators that control fatty acid and endogenous cholesterol production in cells. Several viruses have been reported to regulate intracellular cholesterol synthesis by altering the amount and activity of SREBPs. For instance, increased expression of SREBP1 in hepatocytes of patients with liver steatosis caused by hepatitis B virus infection has been correlated with increased lipid accumulation in liver tissue [64]. It has also been confirmed that HCMV mediates the activation of SREBP1 by inducing PKR-like endoplasmic reticulum kinase in fibroblasts, thereby increasing intracellular adipogenesis [65, 66]. Therefore, in SHEDs, HCMV infection might suppress intracellular cholesterol synthesis by influencing the production and cellular localization of SREBPs in the cytoplasm and nucleus.

The cleavage activation process of SREBPs mainly occurs on the ER and Golgi apparatus. Cleaved SREBPs enter the nucleus and play regulatory roles. In the current study, SREBPs were observed aggregated around the nucleus and multiple discrete small foci appeared in the nucleus after HCMV infection. It was speculated that the perinuclear aggregation of SREBPs found in this study might be caused by following reasons: the cleavage of SREBPs from SCAP was interrupted, preventing SREBPs from leaving the organelles; the entry of cleaved SREBPs into the nucleus was blocked during infection. SREBP1 is responsible for fatty acid synthesis, while SREBP2 mainly participates in the regulation of cholesterol biosynthesis [46]. It was confirmed that the amount of SREBP2 in nucleus was significantly reduced after HCMV infection. Thus, the suppression of intracellular cholesterol biosynthesis was likely a result of the blockade of SREBP2 migration into nucleus.

To address the formation of the discrete small foci of SREBPs within the nucleus, confocal immunofluorescence detection of the viral proteins UL44 and SREBP was performed. Since HCMV UL44 mediates the replication of the viral genome, the intranuclear localization of UL44 can be considered as the location of the viral genome. According to our results, SREBPs and UL44 were not co-localized, so the discrete foci of SREBPs were not due to intensive binding of SREBPs with the HCMV genome. As a result, the mechanism of HCMV infection influencing the activities of SREBPs requires further investigation.

In conclusion, our results elucidate a pathogenic mechanism of neural differentiation disorders, providing new insight into the prevention and treatment of nervous system diseases caused by congenital HCMV infection.

### Electronic supplementary material

Below is the link to the electronic supplementary material.

Supplementary Material 1

Supplementary Material 2

Supplementary Material 3

Supplementary Material 4

Supplementary Material 5

Supplementary Material 6

## Data Availability

The sequencing data were deposited in the Gene Expression Omnibus database under accession number GSE182788.

## Abbreviations

CNS: Central nervous system
DEGs: Differentially expressed genes;
DMEM: Dulbecco’s minimal essential medium;
FC: Free cholesterol;
GAPDH: Glyceraldehyde 3-phosphate dehydrogenase;
GFP: Green fluorescent protein;
GSE: Gene set enrichment analysis;
HCMV: Human cytomegalovirus;
HELFs: Human embryonic lung fibroblasts;
HMGCR: 3-hydroxy-3-methylglutaryl-coenzyme A reductase;
hpi: Hours post infection;
HRP: Horseradish peroxidase;
IE1: Immediate-early 1 protein;
IFA: Immunofluorescence assay;
IPA: Ingenuity Pathway Analysis;
iPSC: Induced pluripotent stem cells;
MAP2: Microtubule-associated protein 2;
MOI: Multiplicities of infection;
MSC: Mesenchymal stem cells;
NES: Normalized enrichment score;
NeuN: Neural nuclei;
NPCs: Neural precursor cells;
NSCs: Neural stem cells;
PBS: Phosphate-bufered saline;
PNS: Peripheral nervous system;
PVDF: Polyvinylidene difluoride;
SCAP: SREBP cleavage activation protein;
SDS-PAGE: SDS-polyacrylamide gel electrophoresis;
SHEDs: Stem cells from human exfoliated deciduous teeth;
SREBP: Sterol regulatory element binding protein;
TC: Total cholesterol;

## References

[1] Malm G, Engman ML (2007). Congenital cytomegalovirus infections. Semin Fetal Neonatal Med.

[2] Cheeran MC, Lokensgard JR, Schleiss MR (2009). Neuropathogenesis of congenital cytomegalovirus infection: disease mechanisms and prospects for intervention. Clin Microbiol Rev.

[3] Nelson CT, Demmler GJ (1997). Cytomegalovirus infection in the pregnant mother, fetus, and newborn infant. Clin Perinatol.

[4] Odeberg J, Wolmer N, Falci S, Westgren M, Seiger A, Söderberg-Nauclér C (2006). Human cytomegalovirus inhibits neural differentiation and induces apoptosis in human neural precursor cells. J Virol.

[5] Odeberg J, Wolmer N, Falci S, Westgren M, Sundtröm E, Seiger A, Söderberg-Nauclér C (2007). Late human cytomegalovirus (HCMV) proteins inhibit differentiation of human neural precursor cells into astrocytes. J Neurosci Res.

[6] Cheeran MC, Hu S, Gekker G, Lokensgard JR (2000). Decreased cytomegalovirus expression following proinflammatory cytokine treatment of primary human astrocytes. J Immunol.

[7] Cheeran MC, Hu S, Yager SL, Gekker G, Peterson PK, Lokensgard JR (2001). Cytomegalovirus induces cytokine and chemokine production differentially in microglia and astrocytes: antiviral implications. J Neurovirol.

[8] Cheeran MC, Hu S, Sheng WS, Peterson PK, Lokensgard JR (2003). CXCL10 production from cytomegalovirus-stimulated microglia is regulated by both human and viral interleukin-10. J Virol.

[9] Cheeran MC, Hu S, Ni HT, Sheng W, Palmquist JM, Peterson PK, Lokensgard JR (2005). Neural precursor cell susceptibility to human cytomegalovirus diverges along glial or neural differentiation pathways. J Neurosci Res.

[10] Liu XJ, Jiang X, Huang SN, Sun JY, Zhao F, Zeng WB, Luo MH (2017). Human cytomegalovirus infection dysregulates neural progenitor cell fate by disrupting Hes1 rhythm and down-regulating its expression. Virol Sin.

[11] Liu XJ, Yang B, Huang SN, Wu CC, Li XJ, Cheng S, Jiang X, Hu F, Ming YZ, Nevels M, Britt WJ, Rayner S, Tang Q, Zeng WB, Zhao F, Luo MH (2017). Human cytomegalovirus IE1 downregulates Hes1 in neural progenitor cells as a potential E3 ubiquitin ligase. PLoS Pathog.

[12] Kriegstein A, Alvarez-Buylla A (2009). The glial nature of embryonic and adult neural stem cells. Annu Rev Neurosci.

[13] Pawolski V, Schmidt MHH (2020). Neuron-Glia Interaction in the developing and adult enteric nervous system. Cells.

[14] Krstanović F, Britt WJ, Jonjić S, Brizić I (2021). Cytomegalovirus infection and inflammation in developing brain. Viruses.

[15] D’Aiuto L, Di Maio R, Heath B, Raimondi G, Milosevic J, Watson AM, Bamne M, Parks WT, Yang L, Lin B, Miki T, Mich-Basso JD, Arav-Boger R, Sibille E, Sabunciyan S, Yolken R, Nimgaonkar V (2012). Human induced pluripotent stem cell-derived models to investigate human cytomegalovirus infection in neural cells. PLoS ONE.

[16] Luo MH, Schwartz PH, Fortunato EA (2008). Neonatal neural progenitor cells and their neural and glial cell derivatives are fully permissive for human cytomegalovirus infection. J Virol.

[17] Belzile JP, Stark TJ, Yeo GW, Spector DH (2014). Human cytomegalovirus infection of human embryonic stem cell-derived primitive neural stem cells is restricted at several steps but leads to the persistence of viral DNA. J Virol.

[18] Chai Y, Jiang X, Ito Y, Bringas P, Han J, Rowitch DH, Soriano P, McMahon AP, Sucov HM (2000). Fate of the mammalian cranial neural crest during tooth and mandibular morphogenesis. Development.

[19] Miura M, Gronthos S, Zhao M, Lu B, Fisher LW, Robey PG, Shi S (2003). SHED: stem cells from human exfoliated deciduous teeth. Proc Natl Acad Sci USA.

[20] Liu Y, Jing H, Kou X, Chen C, Liu D, Jin Y, Lu L, Shi S (2018). PD-1 is required to maintain stem cell properties in human dental pulp stem cells. Cell Death Differ.

[21] Wang J, Wang X, Sun Z, Wang X, Yang H, Shi S, Wang S (2010). Stem cells from human-exfoliated deciduous teeth can differentiate into dopaminergic neuron-like cells. Stem Cells Dev.

[22] Sakai K, Yamamoto A, Matsubara K, Nakamura S, Naruse M, Yamagata M, Sakamoto K, Tauchi R, Wakao N, Imagama S, Hibi H, Kadomatsu K, Ishiguro N, Ueda M (2012). Human dental pulp-derived stem cells promote locomotor recovery after complete transection of the rat spinal cord by multiple neuro-regenerative mechanisms. J Clin Invest.

[23] Sugimura-Wakayama Y, Katagiri W, Osugi M, Kawai T, Ogata K, Sakaguchi K, Hibi H (2015). Peripheral nerve regeneration by secretomes of stem cells from human exfoliated deciduous teeth. Stem Cells Dev.

[24] Zhao F, Shen ZZ, Liu ZY, Zeng WB, Cheng S, Ma YP, Rayner S, Yang B, Qiao GH, Jiang HF, Gao S, Zhu H, Xu FQ, Ruan Q, Luo MH (2016). Identification and BAC construction of Han, the first characterized HCMV clinical strain in China. J Med Virol.

[25] Britt WJ (2010) Human cytomegalovirus: propagation, quantification, and storage. Curr Protoc Microbiol Chap. 14. 10.1002/9780471729259.mc14e03s18 20812216

[26] Martinez Saez D, Sasaki RT, Neves AD, da Silva MC (2016). Stem cells from human exfoliated deciduous teeth: a growing literature. Cells Tissues Organs.

[27] Zhang N, Chen B, Wang W, Chen C, Kang J, Deng SQ, Zhang B, Liu S, Han F (2016). Isolation, characterization and multi-lineage differentiation of stem cells from human exfoliated deciduous teeth. Mol Med Rep.

[28] Ko CS, Chen JH, Su WT (2020). Stem cells from human exfoliated deciduous teeth: a concise review. Curr Stem Cell Res Ther.

[29] Takano T, Tsukiyama-Kohara K, Hayashi M, Hirata Y, Satoh M, Tokunaga Y, Tateno C, Hayashi Y, Hishima T, Funata N, Sudoh M, Kohara M (2005). Augmentation of DHCR24 expression by hepatitis C virus infection facilitates viral replication in hepatocytes. J Hepatol.

[30] Takano T, Endoh M, Fukatsu H, Sakurada H, Doki T, Hohdatsu T (2017). The cholesterol transport inhibitor U18666A inhibits type I feline coronavirus infection. Antiviral Res.

[31] Kong Y, Wu M, Wan X, Sun M, Zhang Y, Wu Z, Li C, Liang X, Gao L, Ma C, Yue X (2023). Lipophagy-mediated cholesterol synthesis inhibition is required for the survival of hepatocellular carcinoma under glutamine deprivation. Redox Biol.

[32] Inoue K, Imai Y (2015). Fatostatin, an SREBP inhibitor, prevented RANKL-induced bone loss by suppression of osteoclast differentiation. Biochim Biophys Acta.

[33] Choi Y, Kawazoe Y, Murakami K, Misawa H, Uesugi M (2003). Identification of bioactive molecules by adipogenesis profiling of organic compounds. J Biol Chem.

[34] Kamisuki S, Mao Q, Abu-Elheiga L, Gu Z, Kugimiya A, Kwon Y, Shinohara T, Kawazoe Y, Sato S, Asakura K, Choo HY, Sakai J, Wakil SJ, Uesugi M (2009). A small molecule that blocks fat synthesis by inhibiting the activation of SREBP. Chem Biol.

[35] Li X, Chen YT, Hu P, Huang WC (2014). Fatostatin displays high antitumor activity in prostate cancer by blocking SREBP-regulated metabolic pathways and androgen receptor signaling. Mol Cancer Ther.

[36] Shao W, Machamer CE, Espenshade PJ (2016). Fatostatin blocks ER exit of SCAP but inhibits cell growth in a SCAP-independent manner. J Lipid Res.

[37] Mizoguchi T, Edano T, Koshi T (2004). A method of direct measurement for the enzymatic determination of cholesteryl esters. J Lipid Res.

[38] Lendahl U, Zimmerman LB, McKay RD (1990). CNS stem cells express a new class of intermediate filament protein. Cell.

[39] Mignone JL, Kukekov V, Chiang AS, Steindler D, Enikolopov G (2004). Neural stem and progenitor cells in nestin-GFP transgenic mice. J Comp Neurol.

[40] Chiereghin A, Turello G, Borgatti EC, Simonazzi G, Felici S, Leone M, Salfi NCM, Santini D, Lazzarotto T (2023). Fetal brain damage in human fetuses with congenital cytomegalovirus infection: histological features and viral tropism. Cell Mol Neurobiol.

[41] Menezes JR, Luskin MB (1994). Expression of neuron-specific tubulin defines a novel population in the proliferative layers of the developing telencephalon. J Neurosci.

[42] Dehmelt L, Halpain S (2005). The MAP2/Tau family of microtubule-associated proteins. Genome Biol.

[43] Duan W, Zhang YP, Hou Z, Huang C, Zhu H, Zhang CQ, Yin Q (2016). Novel insights into NeuN: from neuronal marker to Splicing Regulator. Mol Neurobiol.

[44] Sharpe LJ, Brown AJ (2013). Controlling cholesterol synthesis beyond 3-hydroxy-3-methylglutaryl-CoA reductase (HMGCR). J Biol Chem.

[45] Sharpe LJ, Howe V, Prabhu AV, Luu W, Brown AJ (2015). Navigating the shallows and rapids of cholesterol synthesis downstream of HMGCR. J Nutr Sci Vitaminol (Tokyo).

[46] Eberlé D, Hegarty B, Bossard P, Ferré P, Foufelle F (2004). SREBP transcription factors: master regulators of lipid homeostasis. Biochimie.

[47] Sakakura Y, Shimano H, Sone H, Takahashi A, Inoue N, Toyoshima H, Suzuki S, Yamada N (2001) Sterol regulatory element-binding proteins induce an entire pathway of cholesterol synthesis [published correction appears in Biochem Biophys Res Commun 287(1):311. Inoue K [corrected to Inoue N]]. Biochem Biophys Res Commun 286(1):176–183 10.1006/bbrc.2001.5375 11485325

[48] Qiao GH, Zhao F, Cheng S, Luo MH (2016). Multipotent mesenchymal stromal cells are fully permissive for human cytomegalovirus infection. Virol Sin.

[49] Tsutsui Y (2009). Effects of cytomegalovirus infection on embryogenesis and brain development. Congenit Anom (Kyoto).

[50] Schleiss MR, McVoy MA (2010). Guinea Pig Cytomegalovirus (GPCMV): a model for the study of the Prevention and Treatment of Maternal-Fetal Transmission. Future Virol.

[51] Schleiss MR (2006). Nonprimate models of congenital cytomegalovirus (CMV) infection: gaining insight into pathogenesis and prevention of disease in newborns. ILAR J.

[52] Wu CC, Jiang X, Wang XZ, Liu XJ, Li XJ, Yang B, Ye HQ, Harwardt T, Jiang M, Xia HM, Wang W, Britt WJ, Paulus C, Nevels M, Luo MH (2018). Human cytomegalovirus Immediate Early 1 protein causes loss of SOX2 from neural progenitor cells by Trapping Unphosphorylated STAT3 in the Nucleus. J Virol.

[53] Jiang X, Liu S, Fu YR, Liu XJ, Li XJ, Yang B, Jiang HF, Shen ZZ, Alemu EA, Vazquez P, Tang Y, Kaarbø M, McVoy MA, Rayner S Luo MH 2023 human cytomegalovirus infection perturbs neural progenitor cell fate via the expression of viral microRNAs. J Med Virol 95(2):e28574 10.1002/jmv.28574 PMC12812264 36772841

[54] Niu D, Zhang X, Zhang S, Fan T, Zhou X, Wang H, Zhang X, Nan F, Jiang S, Liu F, Wang Y, Wang B 2023 human cytomegalovirus IE2 disrupts neural Progenitor Development and induces Microcephaly in Transgenic Mouse. Mol Neurobiol 60(7):3883–3897 10.1007/s12035-023-03310-1 PMC10224843 36991278

[55] Weiland KL, Oien NL, Homa F, Wathen MW (1994). Functional analysis of human cytomegalovirus polymerase accessory protein. Virus Res.

[56] Boutolleau D, Deback C, Bressollette-Bodin C, Conan F, Aït-Arkoub Z, Imbert-Marcille BM, Agut H (2009). Genetic analysis and putative role in resistance to antivirals of the human cytomegalovirus DNA polymerase UL44 processivity factor. Antivir Ther.

[57] Kapadia SB, Barth H, Baumert T, McKeating JA, Chisari FV (2007). Initiation of hepatitis C virus infection is dependent on cholesterol and cooperativity between CD81 and scavenger receptor B type I. J Virol.

[58] Waris G, Felmlee DJ, Negro F, Siddiqui A (2020). Hepatitis C virus induces proteolytic cleavage of sterol regulatory element binding proteins and stimulates their phosphorylation via oxidative stress. J Virol.

[59] Low H, Mukhamedova N, Cui HL, McSharry BP, Avdic S, Hoang A, Ditiatkovski M, Liu Y, Fu Y, Meikle PJ, Blomberg M, Polyzos KA, Miller WE, Religa P, Bukrinsky M, Soderberg-Naucler C, Slobedman B, Sviridov D (2016). Cytomegalovirus restructures lipid rafts via a US28/CDC42-Mediated pathway, enhancing cholesterol efflux from Host Cells. Cell Rep.

[60] Sviridov D, Mukhamedova N (2016). Cdc42 - A tryst between host cholesterol metabolism and infection. Small GTPases.

[61] Liu B, Ma Y, Huang Y, Liu Z, Ruan Q, Qi Y (2022). Inhibition of human cytomegalovirus particle maturation by activation of liver X receptor. Front Microbiol.

[62] Mauch DH, Nägler K, Schumacher S, Göritz C, Müller EC, Otto A, Pfrieger FW (2001). CNS synaptogenesis promoted by glia-derived cholesterol. Science.

[63] Suzuki S, Numakawa T, Shimazu K, Koshimizu H, Hara T, Hatanaka H, Mei L, Lu B, Kojima M (2004). BDNF-induced recruitment of TrkB receptor into neural lipid rafts: roles in synaptic modulation. J Cell Biol.

[64] Horton JD, Goldstein JL, Brown MS (2002). SREBPs: activators of the complete program of cholesterol and fatty acid synthesis in the liver. J Clin Invest.

[65] Yu Y, Maguire TG, Alwine JC (2012). Human cytomegalovirus infection induces adipocyte-like lipogenesis through activation of sterol regulatory element binding protein 1. J Virol.

[66] Yu Y, Pierciey FJ, Maguire TG, Alwine JC (2013). PKR-like endoplasmic reticulum kinase is necessary for lipogenic activation during HCMV infection. PLoS Pathog.

