# Supplementary material for: Human cytomegalovirus infection impairs neural differentiation via repressing sterol regulatory element binding protein 2-mediated cholesterol biosynthesis
Source: Cell Mol Life Sci. 2024 Jul 6;81(1):289. doi: 10.1007/s00018-024-05278-0 (PMC11335213; doi:10.1007/s00018-024-05278-0)
Supplement: Supplementary file 1 — Supplementary Material 1 [file 18_2024_5278_MOESM1_ESM.docx]

**Table S1.** Oligonucleotides used in this study.

| Gene Name | Sequence (5’-3’) | Length of Products |
| --- | --- | --- |
| UL123 | Forward: TGCTGTGCTGCTATGTCTTAGAGG | 63bp |
|  | Reverse: TTGGTTATCAGAGGCCGCTTGG |  |
| UL32 | Forward: ACGCCGTCCAGAATACGTAAAGC | 65bp |
|  | Reverse: TTTCTGGCTCGTGGATGTCGTC |  |
| UL55 | Forward: TACGTGCAGTACGGTCAACTGG | 68bp |
|  | Reverse: ACATTCCTCAGTGCGGTGGTTG |  |
| UL44 | Forward: GTGCGCGCCCGATTTCAATATG | 69bp |
|  | Reverse: GCTTTCGCGCACAATGTCTTGA |  |
| UL83 | Forward: TGGAAGAGGACCTGACGATGAC | 78bp |
|  | Reverse: CACAACACCGTAAAGCCGTTGC |  |
| UL99 | Forward: ACTCCGCGCCAAAAGAAGAT | 78bp |
|  | Reverse: TTAAAAGGGCAAGGAGGCGG |  |
| GAPDH | Forward: TGGTGAAGACGCCAGTGGA | 137bp |
|  | Reverse: GCACCGTCAAGGCTGAGAAC |  |
| HMGCR | Forward: TGATTGACCTTTCCAGAGCAAG | 102bp |
|  | Reverse: CTAAAATTGCCATTCCACGAGC |  |
| FDPS | Forward: TGTGACCGGCAAAATTGGC | 75bp |
|  | Reverse: GCCCGTTGCAGACACTGAA |  |
| HSD17B7 | Forward: TGGGATCATGCCTAATCCACA | 188bp |
|  | Reverse: CCAGTTCCCGAATCAGGATAAAA |  |
| MSMO1 | Forward: TGCTTTGGTTGTGCAGTCATT | 143bp |
|  | Reverse: GGATGTGCATATTCAGCTTCCA |  |
| FDFT1 | Forward: CCACCCCGAAGAGTTCTACAA | 134bp |
|  | Reverse: TGCGACTGGTCTGATTGAGATA |  |
| NSDHL | Forward: AAGAGATGCACAGTGATCGGT | 124bp |
|  | Reverse: GCACCTGGGGATTATCAAACC |  |
| SQLE | Forward: TGACAATTCTCATCTGAGGTCCA | 179bp |
|  | Reverse: CAGGGATACCCTTTAGCAGTTTT |  |
| EBP | Forward: CACAGGGGTCTTAGTCGTGAC | 119bp |
|  | Reverse: CCAGGTGAATGAACCCACACA |  |
| TM7SF2 | Forward: GTCGCCTGCGCTATCCTATTA | 178bp |
|  | Reverse: TGCGCCTTCATGTAGAGAAAGA |  |
| DHCR7 | Forward: GCTGCAAAATCGCAACCCAA | 123bp |
|  | Reverse: GCTCGCCAGTGAAAACCAGT |  |
| DHCR24 | Forward: GCCGCTCTCGCTTATCTTCG | 144bp |
|  | Reverse: GTCTTGCTACCCTGCTCCTT |  |
| ACAT1 | Forward: ATGCCAGTACACTGAATGATGG | 157bp |
|  | Reverse: GATGCAGCATATACAGGAGCAA |  |
| ACAT2 | Forward: CTTTAGCACGGATAGTTTCCTGG | 151bp |
|  | Reverse: GCTGCAAAGGCTTCATTGATTTC |  |

**Table S2.**Differently expressed genes related to sterol biosynthesis between neurogenic induced SHEDs with or without HCMV infection.

| Gene ID | log2 Fold Changes | | Gene Name |
| --- | --- | --- | --- |
|  | SHEDi | hSHEDi |  |
| DRD2 | 11.326 | 11.428 | dopamine receptor D2 |
| PRLR | 8.531 | 8.534 | prolactin receptor |
| ALDH1A2 | 7.041 | 8.009 | aldehyde dehydrogenase 1 family member A2 |
| ST8SIA4 | 7.041 | 8.563 | ST8 alpha-N-acetyl-neuraminide alpha-2,8-sialyltransferase 4 |
| C3 | 4.514 | 5.832 | complement C3 |
| BMP6 | 4.441 | 4.514 | bone morphogenetic protein 6 |
| G0S2 | 3.447 | 4.413 | G0/G1 switch 2 |
| PPARGC1A | 3.223 | 5.190 | PPARG coactivator 1 alpha |
| GPR37 | 2.690 | 3.0584 | G protein-coupled receptor 37 |
| APOC1 | 2.491 | 3.181 | apolipoprotein C1 |
| APOE | 2.403 | 3.557 | apolipoprotein E |
| B3GNT5 | 2.347 | 2.479 | UDP-GlcNAc:betaGal beta-1,3-N-acetylglucosaminyltransferase 5 |
| CD74 | 2.329 | 4.409 | CD74 molecule |
| DGAT2 | 2.219 | 3.074 | diacylglycerol O-acyltransferase 2 |
| C1QTNF1 | 2.142 | 2.536 | C1q and TNF related 1 |
| ANGPTL4 | 1.823 | 3.115 | angiopoietin like 4 |
| PLPP2 | 1.612 | 1.980 | phospholipid phosphatase 2 |
| GGT5 | 1.607 | 2.518 | gamma-glutamyltransferase 5 |
| ALDH1A3 | 1.598 | 1.656 | aldehyde dehydrogenase 1 family member A3 |
| BMP2 | 1.575 | 1.649 | bone morphogenetic protein 2 |
| KAT2A | 1.372 | 1.638 | lysine acetyltransferase 2A |
| VAV3 | 8.778 | 7.624 | vav guanine nucleotide exchange factor 3 |
| NTSR1 | 7.602 | 6.381 | neurotensin receptor 1 |
| ACHE | 7.041 | 6.928 | acetylcholinesterase |
| CYP26B1 | 3.992 | 3.579 | cytochrome P450 family 26 subfamily B member 1 |
| PLCB2 | 3.539 | 3.487 | phospholipase C beta 2 |
| ITPKA | 3.220 | 2.312 | inositol-trisphosphate 3-kinase A |
| TNFRSF21 | 3.173 | 2.110 | TNF receptor superfamily member 21 |
| SNCA | 3.008 | 2.401 | synuclein alpha |
| GPAT3 | 2.671 | 2.537 | glycerol-3-phosphate acyltransferase 3 |
| CTSK | 2.555 | 2.101 | cathepsin K |
| HTR2A | 2.532 | 2.335 | 5-hydroxytryptamine receptor 2A |
| KLF4 | 1.992 | 1.556 | Kruppel like factor 4 |
| GNB3 | 1.714 | 1.507 | G protein subunit beta 3 |
| ABHD5 | 1.639 | 1.592 | abhydrolase domain containing 5 |
| CYP27A1 | 1.439 | 1.257 | cytochrome P450 family 27 subfamily A member 1 |
| ASXL3 | 7.200 | ND | ASXL transcriptional regulator 3 |
| APOD | 5.871 | ND | apolipoprotein D |
| RBP1 | 4.357 | ND | retinol binding protein 1 |
| KHK | 3.449 | ND | ketohexokinase |
| FABP3 | 3.058 | ND | fatty acid binding protein 3 |
| MSMO1 | 2.735 | ND | methylsterol monooxygenase 1 |
| HMGCS1 | 2.473 | ND | 3-hydroxy-3-methylglutaryl-CoA synthase 1 |
| TM7SF2 | 2.336 | ND | transmembrane 7 superfamily member 2 |
| B3GALT1 | 2.319 | ND | beta-1,3-galactosyltransferase 1 |
| EBP | 2.250 | ND | EBP cholestenol delta-isomerase |
| ACAT2 | 2.231 | ND | acetyl-CoA acetyltransferase 2 |
| SORL1 | 2.216 | ND | sortilin related receptor 1 |
| ADORA1 | 2.143 | ND | adenosine A1 receptor |
| HSD17B7 | 2.096 | ND | hydroxysteroid 17-beta dehydrogenase 7 |
| ST3GAL5 | 2.040 | ND | ST3 beta-galactoside alpha-2,3-sialyltransferase 5 |
| ST8SIA1 | 2.038 | ND | ST8 alpha-N-acetyl-neuraminide alpha-2,8-sialyltransferase 1 |
| FABP5 | 2.010 | ND | fatty acid binding protein 5 |
| SQLE | 1.997 | ND | squalene epoxidase |
| PLA2R1 | 1.818 | ND | phospholipase A2 receptor 1 |
| FDPS | 1.777 | ND | farnesyl diphosphate synthase |
| GRIN2A | 1.769 | ND | glutamate ionotropic receptor NMDA type subunit 2A |
| GPER1 | 1.764 | ND | G protein-coupled estrogen receptor 1 |
| IDI1 | 1.714 | ND | isopentenyl-diphosphate delta isomerase 1 |
| NPAS2 | 1.662 | ND | neuronal PAS domain protein 2 |
| PLCH2 | 1.603 | ND | phospholipase C eta 2 |
| RAB38 | 1.599 | ND | RAB38, member RAS oncogene family |
| HMGCR | 1.592 | ND | 3-hydroxy-3-methylglutaryl-CoA reductase |
| PIK3R3 | 1.528 | ND | phosphoinositide-3-kinase regulatory subunit 3 |
| ELOVL6 | 1.528 | ND | ELOVL fatty acid elongase 6 |
| INSIG1 | 1.517 | ND | insulin induced gene 1 |
| OSBPL6 | 1.465 | ND | oxysterol binding protein like 6 |
| MVD | 1.452 | ND | mevalonate diphosphate decarboxylase |
| LPIN1 | 1.450 | ND | lipin 1 |
| NSDHL | 1.423 | ND | NAD(P) dependent steroid dehydrogenase-like |
| FDFT1 | 1.375 | ND | farnesyl-diphosphate farnesyltransferase 1 |
| SCD | 1.267 | ND | stearoyl-CoA desaturase |
| DHCR24 | 1.245 | ND | 24-dehydrocholesterol reductase |

ND, no difference.
