# Supplementary figures and images for: Human cytomegalovirus infection impairs neural differentiation via repressing sterol regulatory element binding protein 2-mediated cholesterol biosynthesis
Source: Cell Mol Life Sci. 2024 Jul 6;81(1):289. doi: 10.1007/s00018-024-05278-0 (PMC11335213; doi:10.1007/s00018-024-05278-0)

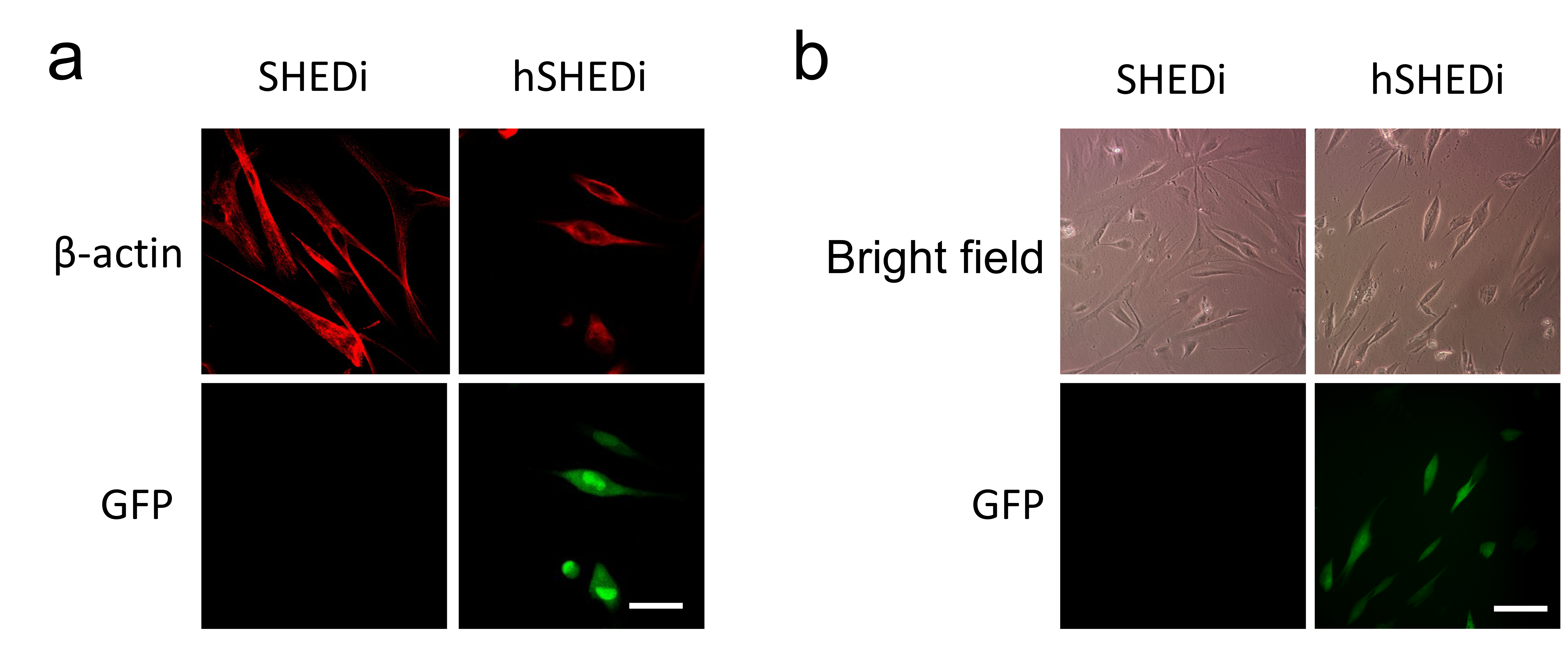

Supplement: Supplementary file 2 — Supplementary Material 2 [file 18_2024_5278_MOESM2_ESM.tif]

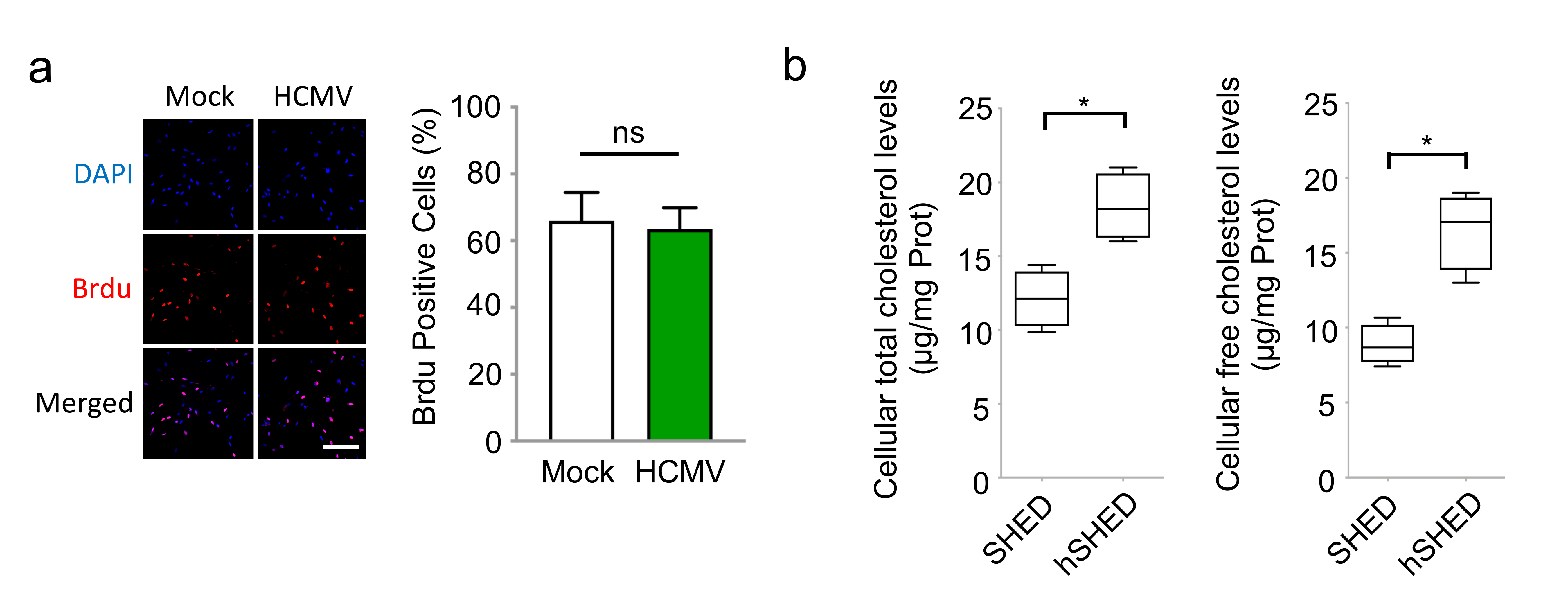

Supplement: Supplementary file 3 — Supplementary Material 3 [file 18_2024_5278_MOESM3_ESM.tif]

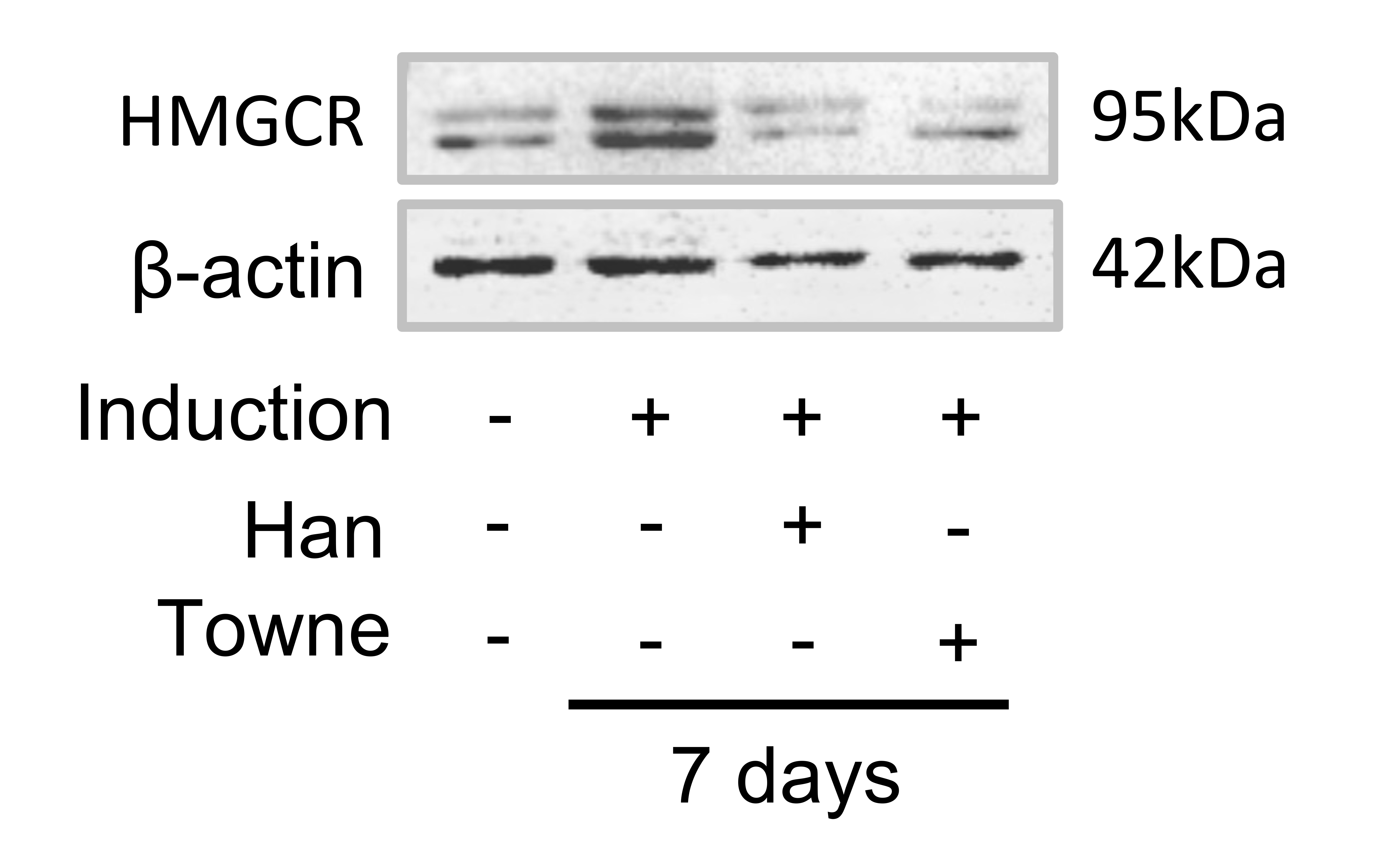

Supplement: Supplementary file 4 — Supplementary Material 4 [file 18_2024_5278_MOESM4_ESM.tif]

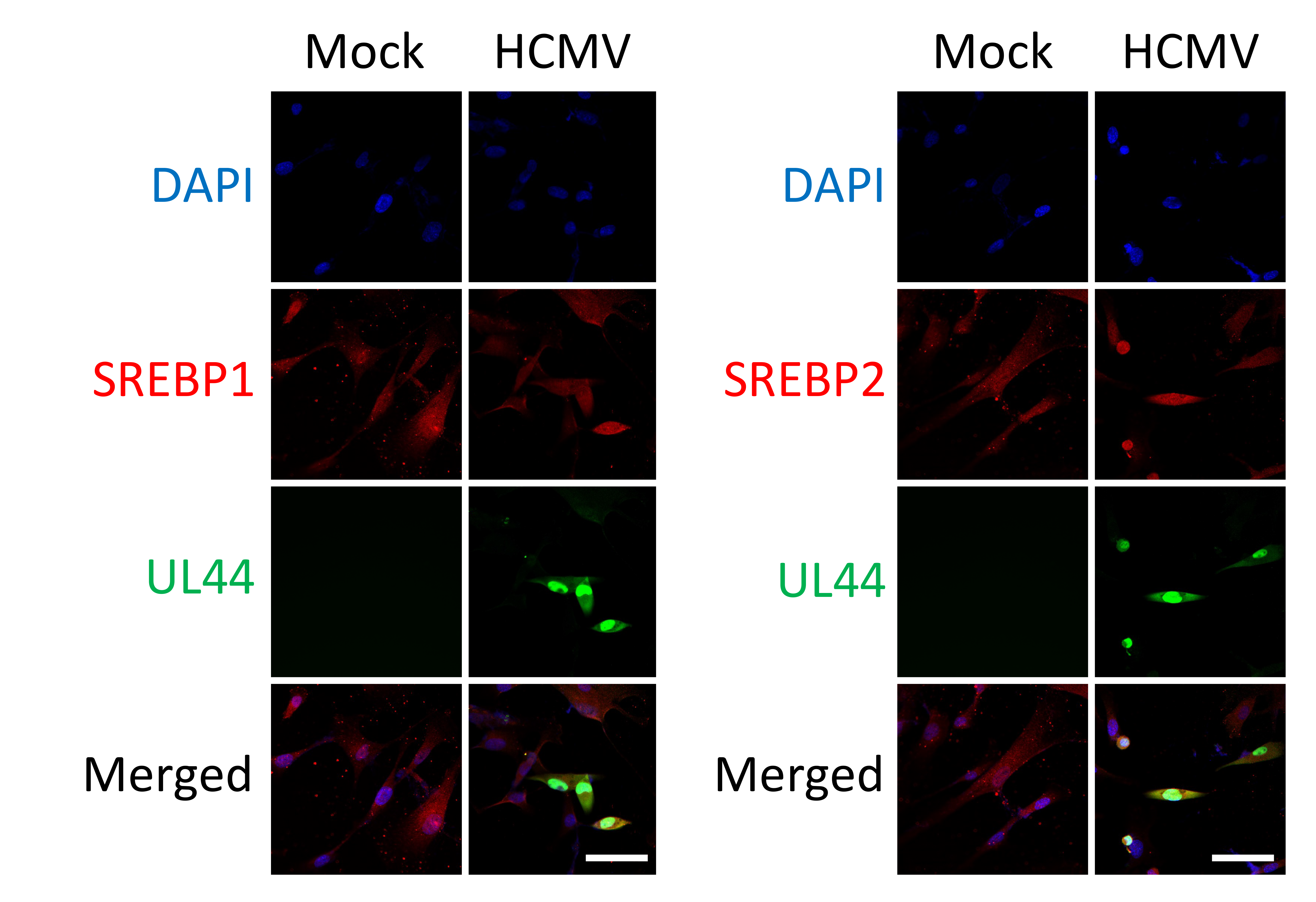

Supplement: Supplementary file 5 — Supplementary Material 5 [file 18_2024_5278_MOESM5_ESM.tif]

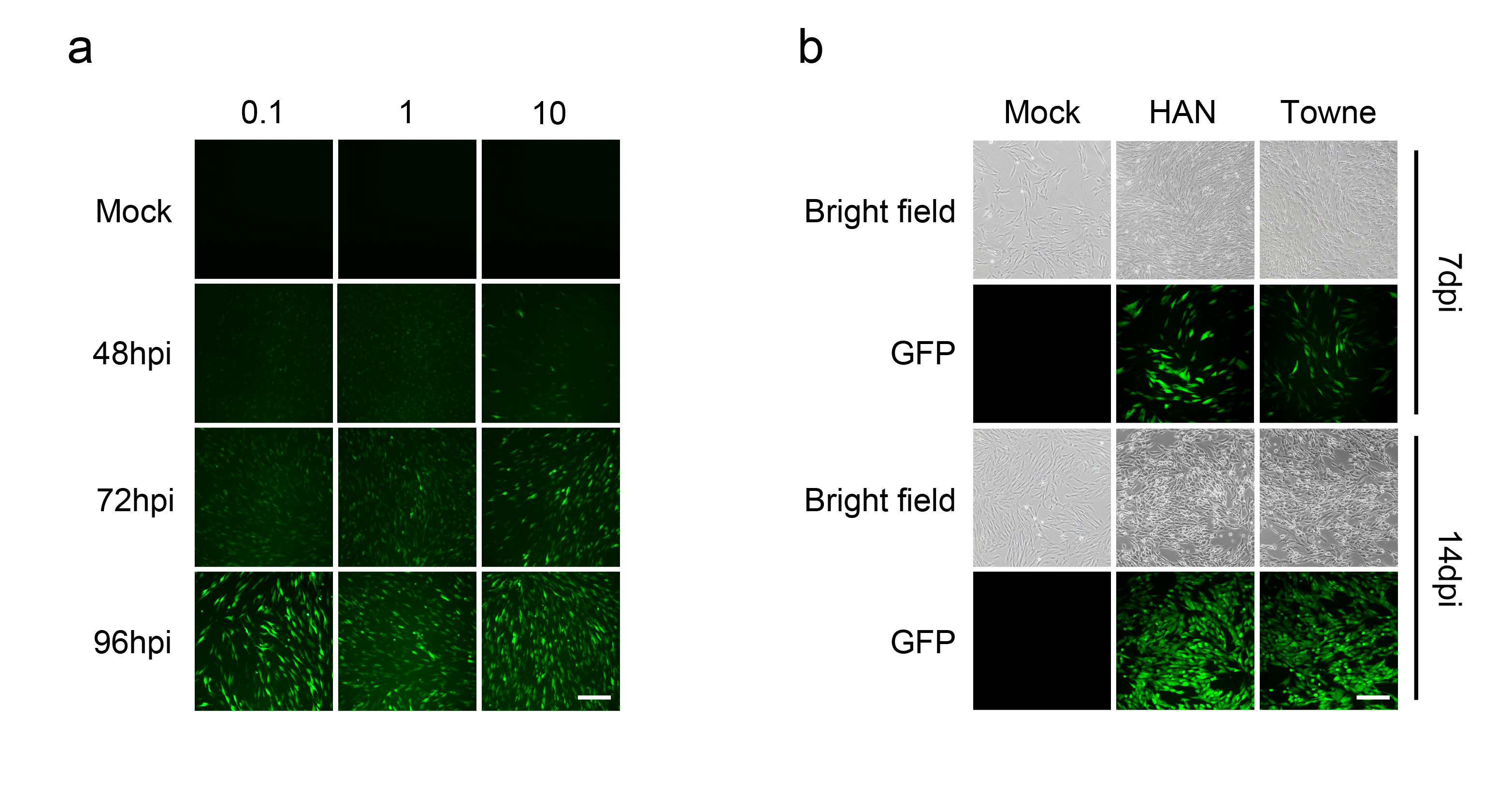

Supplement: Supplementary file 6 — Supplementary Material 6 [file 18_2024_5278_MOESM6_ESM.tif]
